# An Improved Greater Cane Rat Algorithm with Adaptive and Global-Guided Mechanisms for Solving Real-World Engineering Problems

**DOI:** 10.3390/biomimetics10090612

**Published:** 2025-09-10

**Authors:** Yepei Chen, Zhangzhi Tian, Kaifan Zhang, Feng Zhao, Aiping Zhao

**Affiliations:** 1 School of Computer Science, Hubei University of Technology, Wuhan 430068, China; chenyp@whu.edu.cn (Y.C.); 102301179@hbut.edu.cn (Z.T.); 102301178@hbut.edu.cn (K.Z.); 2 Network Information Center, Shijiazhuang University, Shijiazhuang 050035, China; 3 School of Journalism & Communication, Shijiazhuang University, Shijiazhuang 050035, China

**Keywords:** greater cane rat algorithm, adaptive and global-guided mechanisms, solving optimization problems

## Abstract

This study presents an improved variant of the greater cane rat algorithm (GCRA), called adaptive and global-guided greater cane rat algorithm (AGG-GCRA), which aims to alleviate some key limitations of the original GCRA regarding convergence speed, solution precision, and stability. GCRA simulates the foraging behavior of the greater cane rat during both mating and non-mating seasons, demonstrating intelligent exploration capabilities. However, the original algorithm still faces challenges such as premature convergence and inadequate local exploitation when applied to complex optimization problems. To address these issues, this paper introduces four key improvements to the GCRA: (1) a global optimum guidance term to enhance the convergence directionality; (2) a flexible parameter adjustment system designed to maintain a dynamic balance between exploration and exploitation; (3) a mechanism for retaining top-quality solutions to ensure the preservation of optimal results.; and (4) a local perturbation mechanism to help escape local optima. To comprehensively evaluate the optimization performance of AGG-GCRA, 20 separate experiments were carried out across 26 standard benchmark functions and six real-world engineering optimization problems, with comparisons made against 11 advanced metaheuristic optimization methods. The findings indicate that AGG-GCRA surpasses the competing algorithms in aspects of convergence rate, solution precision, and robustness. In the stability analysis, AGG-GCRA consistently obtained the global optimal solution in multiple runs for five engineering cases, achieving an average rank of first place and a standard deviation close to zero, highlighting its exceptional global search capabilities and excellent repeatability. Statistical tests, including the Friedman ranking and Wilcoxon signed-rank tests, provide additional validation for the effectiveness and importance of the proposed algorithm. In conclusion, AGG-GCRA provides an efficient and stable intelligent optimization tool for solving various optimization problems.

## 1. Introduction

Recently, influenced by the collective behaviors observed in biological populations, swarm intelligence-driven metaheuristic optimization algorithms have shown remarkable adaptability and versatility in addressing intricate optimization challenges [1,2,3,4,5]. By simulating behaviors such as co-evolution [6], foraging strategies [7,8,9], or social interactions of biological populations [10,11,12], these algorithms can efficiently perform global searches and local development in complex search spaces, including high-dimensional [13,14,15,16], multimodal, and nonlinear ones [17,18,19,20,21,22]. The core principles of biomimetics—drawing on the self-organization, self-adaptation, and collaboration mechanisms of natural organisms—provide a rich theoretical foundation and practical guidance for the design of optimization algorithms [23]. This has led to their widespread application and in-depth research in fields such as engineering optimization, machine learning parameter tuning, structural design, and robotic control [24,25,26,27,28,29]. For example, the particle swarm optimization (PSO) algorithm is influenced by the collective foraging behaviors of bird and fish groups [30]; the ant colony optimization (ACO) algorithm relies on the pheromone-based navigation of ants [31]; the butterfly optimization algorithm (BOA) is inspired by butterflies’ innate foraging and mating patterns [32]; the whale optimization algorithm (WOA) emulates the bubble-net hunting technique of humpback whales [33]; and the goose optimization algorithm (GOOSE) mirrors the V-shaped formation and coordinated navigation seen during goose migration [34]. These algorithms, by simulating collective behaviors in animal populations, have enabled the efficient exploration and exploitation of complex search spaces, thereby advancing fields such as engineering optimization.

However, these bio-inspired algorithms often face challenges, including slow convergence rates, suboptimal solution accuracy, and limited stability, frequently resulting in premature convergence to local optima. To address these issues, various improvements have been proposed by researchers. For example, Cao et al. [35] developed a global-best guided phase based optimization (GPBO) algorithm, incorporating a global optimum guidance term to enhance convergence directionality in large-scale optimization problems. Yang et al. [36] introduced a spatial information sampling strategy for adaptive parameter control in meta-heuristic algorithms, dynamically adjusting parameters based on the spatial distribution of solutions. Wu et al. [37] enhanced PSO with an elite retention mechanism to improve convergence performance and avoid premature local optima in solving the flexible job shop scheduling problem. Öztaş and Tuş [38] developed a hybrid iterated local search algorithm that incorporates a local perturbation mechanism to explore diverse regions of the search space and improve solutions for the vehicle routing problem with simultaneous pickup and delivery.

The greater cane rat algorithm (GCRA), a recently introduced biologically inspired method, emulates the foraging behavior of the greater cane rat during both mating and non-mating periods, integrating global search and local development features [39]. In the GCRA framework, the greater cane rat’s behavior is modeled in two distinct phases: the non-mating foraging phase and the mating period. During the non-mating phase, the individuals forage independently while sharing indirect information about promising food sources, which, in the algorithm, corresponds to global exploration of the search space. In the mating phase, individuals tend to cluster and compete for mates, representing intensified local search around high-quality areas. These biological strategies are mathematically encoded in position-updating equations, where movement decisions are guided by both random exploration and attraction toward elite individuals, thus translating observed animal behaviors into optimization processes. However, despite its good performance in certain optimization problems, GCRA still faces challenges in high-dimensional, multimodal, and constrained optimization problems, including slow convergence, limited solution precision, and a tendency to become trapped in local optima, restricting its practical application and value.

In order to improve the performance of the GCRA, this paper proposes the adaptive and global-guided greater cane rat algorithm (AGG-GCRA). This algorithm integrates four key improvements into the original GCRA framework, systematically enhancing its convergence, accuracy, and stability. First, AGG-GCRA introduces a global guidance term that incorporates historical best solution information into the individual position update process [40], effectively improving the population’s convergence direction toward the optimal solution region and enhancing its global search ability. Second, an adaptive parameter adjustment mechanism is designed, enabling key parameters to dynamically change during the iteration process, thus balancing exploration and exploitation. Third, an elite retention mechanism periodically retains the current best individual to prevent the loss of high-quality solutions due to random operations, thereby improving the stability and repeatability of the results. Finally, a local perturbation mechanism is introduced to apply minor disturbances to some high-quality individuals [41], enhancing population diversity and assisting the algorithm in avoiding local optima, thereby improving global optimization effectiveness.

To comprehensively evaluate the optimization performance of AGG-GCRA, comprehensive comparative experiments were performed using 26 standard benchmark functions and six practical engineering optimization problems. The results were evaluated against 11 other leading metaheuristic algorithms, such as PSO, WOA, and GOOSE. The experimental results show that AGG-GCRA outperforms the comparison algorithms in terms of convergence speed, solution accuracy, and robustness, particularly in demonstrating lower standard deviations in engineering cases, reflecting its excellent stability and repeatability. Additionally, the statistical significance of the proposed algorithm’s advantages was confirmed using Friedman ranking [42,43] and Wilcoxon signed-rank tests [44,45].

In conclusion, AGG-GCRA addresses the shortcomings of the original GCRA, exhibiting robust global search, local exploitation, and stable convergence, thus establishing it as an effective and dependable optimization tool for a range of complex problems. The primary contributions of this paper are as follows:

- A new greater cane rat optimization algorithm (AGG-GCRA) is introduced, incorporating adaptive mechanisms and global guidance techniques to enhance the algorithm’s convergence rate, solution precision, and stability.
- A comprehensive comparison of AGG-GCRA with 11 mainstream metaheuristic algorithms on 26 benchmark functions shows that AGG-GCRA surpasses the other algorithms in terms of optimization precision, convergence efficiency, and robustness, with its significance confirmed through statistical tests.
- AGG-GCRA was applied to six practical engineering optimization problems, with the algorithm consistently producing high-quality solutions in multiple independent runs, demonstrating its practical value and reliability in engineering applications.

The structure of the paper is organized as follows: Section 2 introduces the basic principles and mechanisms of the original GCRA; Section 3 provides a detailed description of the proposed AGG-GCRA; Section 4 presents experimental comparisons and analyses of AGG-GCRA and 11 comparison algorithms on standard test functions and practical engineering optimization problems; Section 5 concludes the paper and outlines directions for future research.

## 2. Greater Cane Rat Algorithm

The GCRA is a novel metaheuristic optimization technique drawn from the foraging and social behaviors of the African greater cane rat (scientific name: Thryonomys swinderianus). This section elucidates the biological inspiration, mathematical model, and algorithm structure of the GCRA.

### 2.1. Biological Inspiration and Conceptual Model

The African greater cane rat, native to sub-Saharan Africa, has a distinct social structure and foraging behavior, which inspired the design of the GCRA. In its social system, a dominant male leads a group consisting of several females and juveniles. Greater cane rats forage at night, with the males memorizing paths to food and water sources and guiding the group members to them. During the mating season, males and females separate into groups and focus their activities around abundant food areas, thus enhancing foraging efficiency.

**Figure 1:**
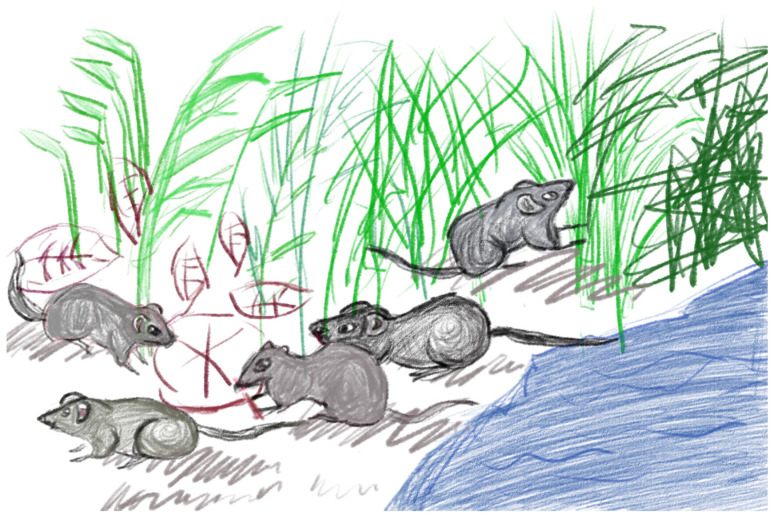
Natural habitat of the greater cane rat.

Figure 1 illustrates the natural habitat of the African greater cane rat. These rats are excellent swimmers, often using water to avoid danger, and are quick and agile on land. While primarily nocturnal, they may also be active during the day. They reside in a matriarchal society, governed by a dominant male, and typically nest in dense vegetation or occasionally in burrows vacated by other animals or termites. When threatened, they either produce a grunting sound or swiftly escape into the water for protection. The shaded region in the figure indicates the water source, with tall reeds growing nearby. The white regions and trails denote the routes to known food sources.

These biological traits are transformed into the exploration, exploitation, and information-sharing mechanisms within the algorithm, which simulate the global and local search processes within the search space.

### 2.2. Population Initialization

The optimization process of the GCRA begins with the random generation of a cane rat population P, which consists of M individuals, each representing a candidate solution located at a specific position in a D-dimensional search space. The population initialization formula is shown as Equation (1):

(1) $$P=\left[\begin{array}{cccc} p_{1,1} & p_{1,2} & \ldots & p_{1,D}\\ p_{2,1} & p_{2,2} & \ldots & p_{2,D}\\ \vdots & \vdots & \ddots & \vdots \\ p_{M,1} & p_{M,2} & \ldots & p_{M,D} \end{array}\right]$$

where the position of the m-th individual in the k-th dimension is determined by Equation (2):

(2) $$p_{m,k}=\lambda \times (U_{k}-L_{k})+L_{k}$$

where λ∈[0,1] is a random number uniformly distributed, and $U_{k}$ and $L_{k}$ represent the upper and lower bounds of the k-th dimension, respectively. This initialization guarantees that the population is evenly spread across the search space, which, in turn, enhances the algorithm’s diversity and exploration capacity.

**Figure 2:**
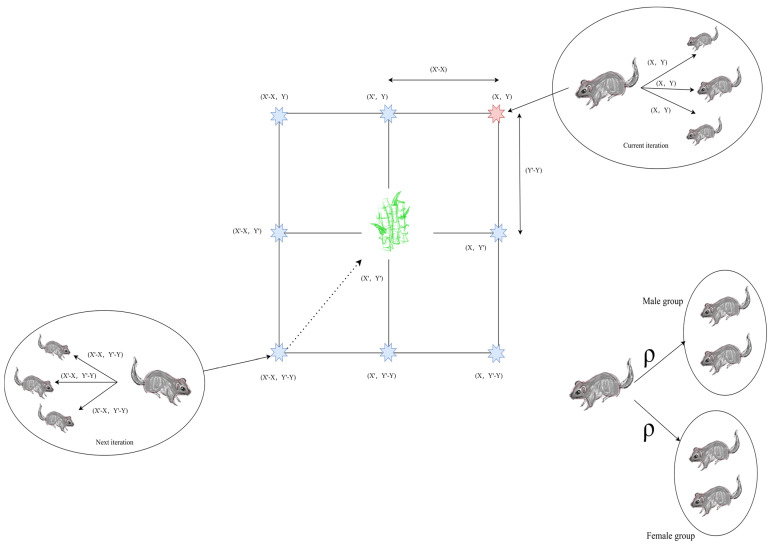
2D possible position vectors.

Figure 2 presents the conceptual framework of the GCRA. Assuming the target food resource is located at coordinates $\left(X^{\prime },Y^{\prime }\right)$, the path to this location is initially identified by the dominant male individual within the group, which then disseminates the corresponding path information to other members, enabling them to adjust their positions accordingly. Positioned at (X,Y), the dominant individual possesses knowledge of the target location $\left(X^{\prime },Y^{\prime }\right)$ and explores several accessible neighboring positions, as guided by Equations (4) and (5). In subsequent iterations, the dominant individual located at the relative position $\left(X^{\prime }-X,\;Y^{\prime }-Y\right)$ continues to execute similar path update procedures. During the breeding season, information regarding food resource locations is shared across the group, prompting the population to segregate into male and female subsets. Each subset migrates toward food-rich regions and establishes provisional campsites in those areas.

### 2.3. Behavioral Modeling and Position Update

The GCRA alternates between two main phases based on the control parameter α (representing the environmental shifts between the rainy and dry seasons): the exploration phase and the exploitation phase.

In the exploration phase (path construction period), cane rat individuals either explore unknown areas or follow the paths remembered by the dominant male. The fundamental position update rule is shown as Equation (3):

(3) $$q_{m,k}^{new}=0.7\times \frac{q_{m,k}+q_{r,k}}{2}$$

where $q_{m,k}$ denotes the position of the m-th individual in the k-th dimension, and $q_{r,k}$ is the position of another random individual in the same dimension. To enhance the update strategy, three additional coefficients are introduced as Equations (4)–(6):

(4) $$r=F_{q_{r}}-\left(\frac{t\times F_{q_{r}}}{T_{max}},\right)$$

(5) α=2r×ξ−r,

(6) β=2r×η−r,

where $F_{q_{r}}$ is the fitness value of individual r, t and $T_{max}$ are the current iteration and maximum iteration count, and ξ and η are random variables uniformly distributed in the range [0,1].

In the exploitation phase (mating season foraging period), males and females search independently, focusing on approaching the position of a randomly selected female to accelerate the local search. The position update formula is shown as Equation (7):

(7) $$q_{m,k}^{new}=q_{m,k}+C\times (q_{r,k}-\mu \times q_{f,k})$$

where $q_{f,k}$ represents the position of the randomly selected female individual, μ simulates the offspring count for each female rat, with possible values in the set {1,2,3,4}, and C is the weight parameter controlling the step size.

In addition, the original formulation of the GCRA also defines an alternative exploitation variant, expressed as Equation (8):

(8) $$q_{\left(m,k\right)}^{new}=q_{\left(m,k\right)}+C\times \left(\begin{array}{c} q_{\left(k,k\right)}-\mu \times q_{\left(m,k\right)} \end{array}\right)$$

After generating a candidate position $q_{\left(m,k\right)}^{new}$, the update is only accepted if the new position yields a better fitness; otherwise, the previous position is retained, as shown in Equation (9):

(9) $$if\;F\left(q_{\left(m,k\right)}^{new}\right)<F\left(q_{\left(m,k\right)}\right),q_{\left(m,k\right)}\leftarrow q_{\left(m,k\right)}^{new};\mathrm{else},\;\mathrm{it}\;\mathrm{remain}s\;\mathrm{unchanged}.$$

### 2.4. Dominant Male Selection and Fitness Evaluation

In the GCRA, the dominant male is the individual with the highest fitness in the current population, symbolizing the optimal solution. Other individuals modify their strategies accordingly. Other individuals adjust their strategies based on the position information of the dominant male, thereby optimizing their search direction. The dominant male is updated whenever a better solution emerges in the population, which can be formulated as Equation (10):

(10) $$k=arg\;\underset{i}{min}\;F(q_{i})$$

where k denotes the index of the newly selected dominant male, $arg\;\underset{i}{min}$ represents the operation of finding the index i that yields the minimum value of $F(q_{i})$ among all individuals, and $F(q_{i})$ refers to the fitness value of individual i.

In each iteration, if a better solution is found, the dominant male is updated to ensure the algorithm progresses toward the global optimum. If no better solution is found, the position is updated or adjusted according to the current phase rules to avoid convergence to local optima.

The importance of the GCRA lies not merely in its novelty but in its unique balance between exploration and exploitation inspired by the greater cane rat’s real-world adaptive foraging strategies. Unlike many generic swarm-based metaheuristics, the GCRA integrates memory-based path following and seasonal behavior switching, which naturally encode both long-range search and localized intensification. These mechanisms address the frequent imbalance between global and local search observed in conventional metaheuristics. Furthermore, the separation of male and female subgroups during exploitation phases introduces a structured diversity preservation mechanism, reducing the risk of premature convergence and enhancing performance in complex multimodal optimization landscapes. This combination of behavioral realism and search efficiency underpins its relevance and value for both theoretical study and practical applications.

**Figure 3:**
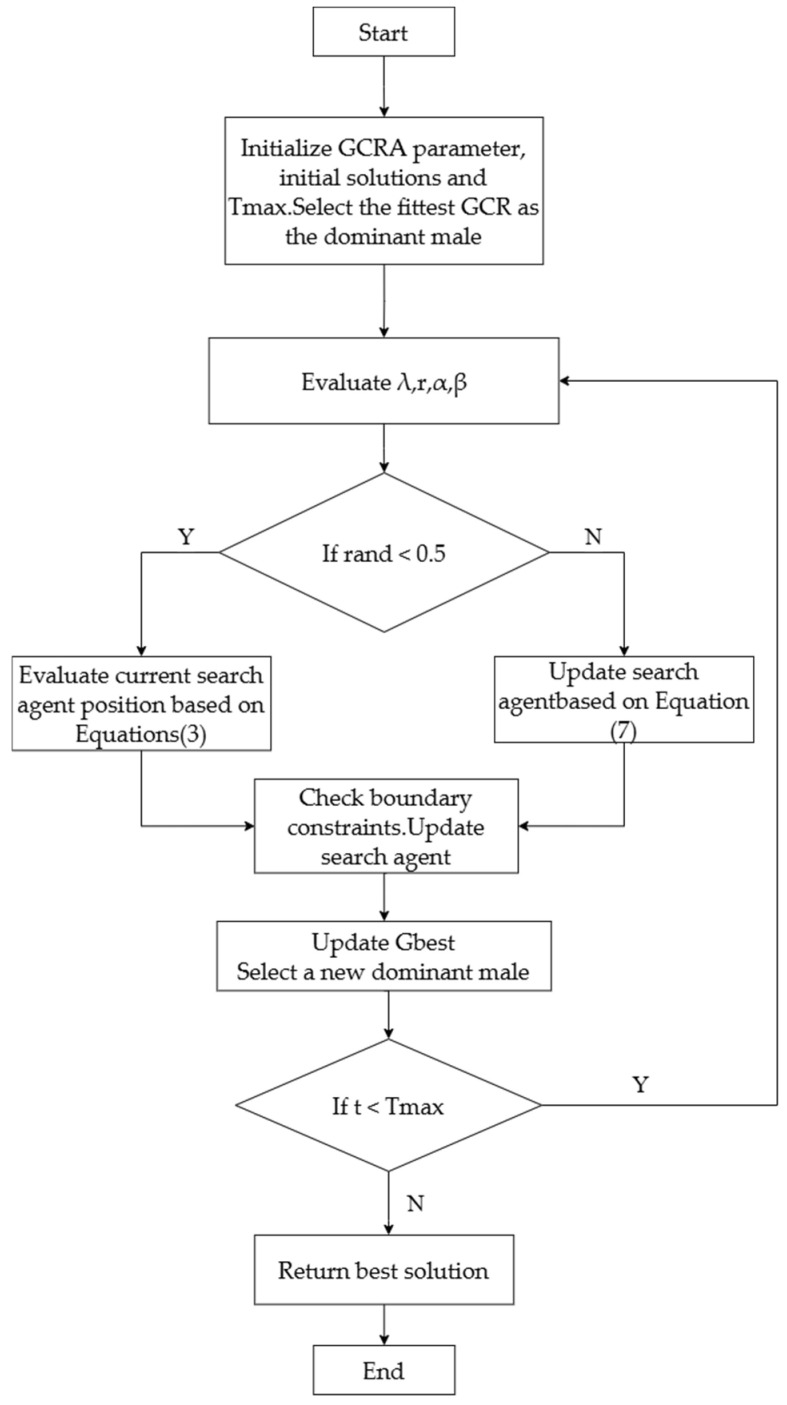
Flowchart of the GCRA.

The algorithm flow of the GCRA is shown in Figure 3.

## 3. Adaptive and Global-Guided Greater Cane Rat Algorithm

To improve the global search capability, local exploitation ability, and convergence stability of the GCRA in addressing complex optimization challenges, this paper introduces an enhanced algorithm, AGG-GCRA, which integrates adaptive global guidance and elite mechanisms. By introducing a global optimum guidance term, an adaptive parameter adjustment strategy, an elite retention mechanism, and a local perturbation mechanism, the AGG-GCRA effectively addresses the issues in the GCRA, including the tendency to become trapped in local optima and slow convergence.

This section highlights the main enhancements of the AGG-GCRA and offers an in-depth description of the four fundamental mechanisms.

### 3.1. Global Optimum Guidance Term

To enhance the convergence precision and search efficiency of the GCRA, a global optimum guidance term is introduced by modifying the original position update formula (Equation (3)), adding a guidance component from the global best position $q_{best}$. In each iteration, the individual position update is influenced not only by a randomly selected individual but also by the current global optimum solution $q_{best}$, as shown in Equation (11):

(11) $$q_{m,k}^{new}=q_{m,k}+\lambda \cdot (q_{best,k}-q_{m,k})$$

where λ is the global guidance learning rate parameter, which dynamically adjusts the influence of the global best solution on individual position updates based on the iteration number and population state. This adjustment improves the convergence directionality while effectively preventing premature convergence. The dynamic mechanism helps maintain population diversity and avoids stagnation.

### 3.2. Adaptive Parameter Adjustment

In order to maintain a dynamic balance between exploration and exploitation, an adaptive parameter adjustment strategy is proposed. The adaptive factor σ(t) is defined as Equation (12):

(12) $$\sigma (t)=\alpha_{0}\times \left(1-\frac{t}{T_{max}}\right)\times (1-\delta )$$

where $\alpha_{0}$ is the initial balance factor, t is the current iteration, $T_{max}$ is the maximum number of iterations, and δ is the relative ratio of the current best solution’s fitness fluctuation to the historical best.

Based on the value of σ(t), the algorithm dynamically decides the proportion of exploration and exploitation formulas to be used: when σ(t)>0.5, exploration operations are prioritized; otherwise, exploitation operations are prioritized. This strategy enhances the algorithm’s search adaptability across different stages.

### 3.3. Elite Preservation Mechanism

To ensure that high-quality solutions in the population are not disrupted by random updates, an elite preservation mechanism is embedded as an additional position update step extending Equation (7). The top 20% of individuals are selected to form an elite set E, and the average solution ${\bar{q}}_{E}$ of these elite individuals is used to guide the position updates of regular individuals as Equation (13):

(13) $$q_{m,k}^{new}=q_{m,k}+\gamma \cdot ({\bar{q}}_{E,k}-q_{m,k})$$

where the adaptive learning rate γ is defined as Equation (14):

(14) $$\gamma =\gamma_{0}\cdot \left(1-\frac{t}{T_{max}}\right)$$

The learning rate γ decreases linearly as the iterations progress, enabling the algorithm to gradually shift from exploration to exploitation. Moreover, using the average position of the elite subset rather than solely relying on the global best individual enhances the algorithm’s robustness by preventing overdependence on a single solution and better maintaining population diversity. This mechanism helps individuals converge toward high-quality regions, improving the accuracy of later-stage searches.

### 3.4. Local Perturbation Strategy

To enhance the algorithm’s capability to avoid becoming trapped in local optima, a local perturbation strategy is introduced. The updated position of an individual incorporates a perturbation term Δ, expressed as Equation (15):

(15) $$q_{m,k}^{new}=q_{m,k}^{new}+\omega \cdot \Delta$$

where Δ follows a mixed distribution as Equation (16):

(16) $$\Delta =\theta_{1}\cdot N(0,\sigma_{1}^{2})+\theta_{2}\cdot \mathrm{Lévy}(\beta )$$

where $N(0,\sigma_{1}^{2})$ represents a normal distribution perturbation, and Lévy(β) represents a Lévy flight distribution. $\theta_{1}+\theta_{2}=1$ is the perturbation weight.

The Lévy flight component introduces a probability-driven mechanism for generating occasional long-distance jumps, which follows a heavy-tailed distribution. This allows the algorithm to escape from local optima by occasionally exploring far regions of the search space that are inaccessible to purely Gaussian perturbations. The combination of Gaussian perturbations for fine-tuning and Lévy flights for large-scale jumps strikes a balance between local exploitation and global exploration, ensuring that the search process remains diverse while improving the chances of finding better solutions. This combines Gaussian noise and Lévy flight for fine-tuned local search and occasional long jumps, acting as a stochastic refinement after the main update steps (modifying Equations (3) and (7)), which enhances the local search capability while maintaining global exploration.

### 3.5. Complexity Analysis

The AGG-GCRA consists mainly of two stages: population initialization and iterative updates. During initialization, the algorithm generates random initial positions for each search agent and dimension, resulting in a time complexity of O(Agents×dimension). In the main loop, during each iteration, the algorithm updates the positions and evaluates the fitness of all agents across the dimensions. The total number of iterations is $T_{max}$, and the overall time complexity is mainly determined by this part. Elite replacement and local perturbation operations are relatively small in comparison and can be ignored. Thus, the time complexity of the algorithm is shown as Equation (17):

(17) $$O(T_{max}\times Agents\times dimension)$$

where $T_{max}$ is the maximum number of iterations, Agents is the population size, and dimension is the dimensionality of the problem. If the objective function is computationally intensive, the time complexity will rise accordingly. More specifically, the position update stage involves vector operations with a computational cost of O(dimension) per agent, while the fitness evaluation stage typically incurs the highest cost at $O(f_{eval})$ per agent, where $O(f_{eval})$ denotes the time complexity of the objective function. Consequently, the full computational complexity can be expressed as $O(T_{max}\times Agents\times (dimension+f_{eval}))$. In cases where $f_{eval}$ dominates, this term becomes the primary contributor to runtime, whereas, for simpler objective functions, the algorithm’s complexity is effectively linear in both the population size and problem dimension.

### 3.6. Summary of the AGG-GCRA

This section systematically presents the core improvement strategies of the AGG-GCRA, covering the four aspects of global optimum guidance, adaptive parameter adjustment, elite retention, and local perturbation. These strategies maintain a well-balanced approach between global exploration and local refinement throughout the search process. By dynamically regulating the transition between exploration and exploitation phases, the algorithm’s adaptability and robustness are improved. The elite set effectively prevents premature convergence, while the local perturbation mechanism improves population diversity, ensuring the algorithm’s capability to avoid local optima in complex search spaces. Overall, the AGG-GCRA achieves multi-level optimization over the original GCRA, accelerating convergence speed and improving the stability and accuracy of the final solution.

**Figure 4:**
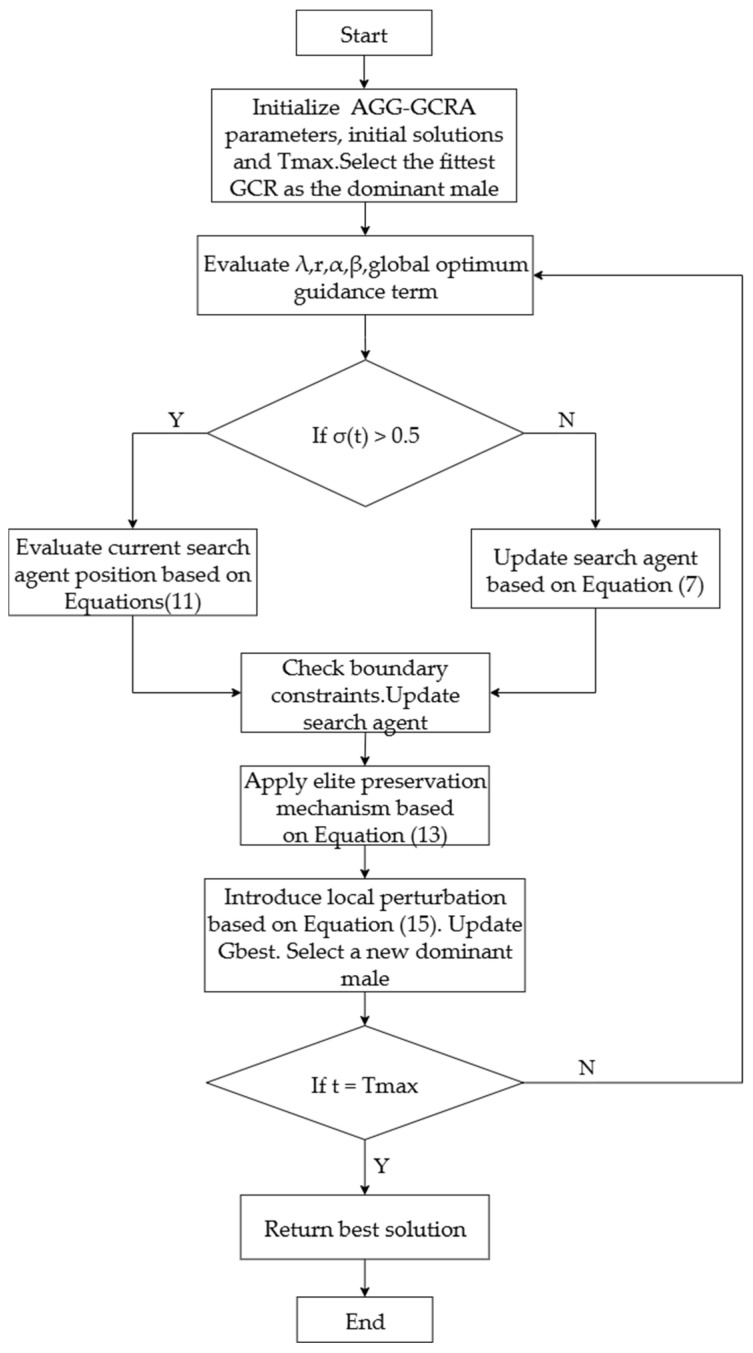
Flowchart of the AGG-GCRA.

To facilitate understanding and practical implementation, Algorithm 1 provides a detailed pseudocode for the AGG-GCRA, clearly illustrating the execution logic of each key step. Additionally, the algorithm flowchart in Figure 4 offers a visual representation of the operational framework. The algorithm is well structured and scientifically designed, providing a solid foundation for performance verification and application in subsequent sections.
**Algorithm 1.** AGG-GCRAInput: Population size M, maximum iterations T_max, parameters α_0_, γ_0_, λ, σ_1_^2^, β, θ_1_, θ_2_, ω
Output: Optimal solution q_best
1. Initialize population positions qₘ (m = 1, 2, …, M)
2. Evaluate fitness f(qₘ) for each individual
3. Set q_best as the global best solution
4. For t = 1 to T_max do
  4.1 Adaptive Parameter Adjustment — (Equation (12))
    Compute σ(t) = α_0_ × (1 - t / T_max) × (1 − δ) (12)
  4.2 For m = 1 to M do
    If σ(t) > 0.5 then // Exploration Phase
      Global Optimum Guidance Term — (Equation (11))
      qₘ,ₖⁿᵉʷ = qₘ,ₖ + λ × (q_best,ₖ − qₘ,ₖ) (11)
    Else // Exploitation Phase
      Elite Preservation Mechanism — (Equation (13)–(14))
      Find top 20% individuals → elite set E
      Compute elite mean position q̄_E
      Adaptive learning rate: γ = γ_0_ × (1 − t / T_max) (14)
      Update position: qₘ,ₖⁿᵉʷ = qₘ,ₖ + γ × (q̄_E,ₖ − qₘ,ₖ) (13)
    End If
    Local Perturbation Strategy—(Equation (15)–(16))
      Generate perturbation: Δ = θ_1_ × N(0, σ_1_^2^) + θ_2_ × Lévy(β) (16)
      Apply perturbation: qₘ,ₖⁿᵉʷ = qₘ,ₖⁿᵉʷ + ω × Δ (15)
    Evaluate fitness of qₘⁿᵉʷ and update qₘ if better
  End For
  Update elite set E and global best q_best
End For
Input: Population size M, maximum iterations T_max, parameters α_0_, γ_0_, λ, σ_1_^2^, β, θ_1_, θ_2_, ω

## 4. Results and Analytical Evaluation of the Experiment

To validate the performance and effectiveness of the proposed AGG-GCRA, this section performs a comparative analysis from two perspectives: standard benchmark functions and practical engineering problems. Specifically, it consists of (1) a comparison with 11 mainstream optimization algorithms on 26 classic benchmark functions, including the GCRA, BOA, WOA, GOOSE, AOA [46], PSO, DE [47], ACO, NSM-BO [48], PSOBOA [49], and FDB-AGSK [50]. The sources of the algorithms are provided in the Appendix A. The parameter configurations for each algorithm can be found in Table 1. For fairness, parameters commonly used across algorithms—such as maximum iterations, population size, and termination conditions—were set according to widely accepted values reported in previous studies, while algorithm-specific parameters were further refined through experimental tuning. This tuning process involved evaluating multiple parameter combinations on representative benchmark functions and selecting those that consistently produced high average performance. Such adjustment of literature-based values ensures that each algorithm performs reasonably and competitively. In Table 1, the term “Agents” denotes the population size, meaning the number of candidate solutions maintained by the algorithm at each iteration; (2) an application verification on six typical engineering optimization problems, followed by a performance analysis compared to the aforementioned algorithms.

Experimental setup: The proposed AGG-GCRA, along with other metaheuristic methods, was implemented in MATLAB 2023a. All tests were carried out on a Windows 10 platform with an Intel(R) Core(TM) i9-14900KF processor (3.10 GHz) and 32 GB of RAM.

### 4.1. Tests on 26 Benchmark Functions

To thoroughly assess the performance of the proposed AGG-GCRA, this paper selects 26 classic test functions as the benchmark set [51,52,53,54,55,56]. These test functions are widely used in metaheuristic optimization, because they effectively reflect the characteristics and difficulty of various optimization problems, making them a crucial tool for validating algorithm performance.

In accordance with the benchmarking guidelines outlined by Beiranvand et al. [57], and aligned with the principles emphasized by Piotrowski et al. [58,59], all experiments were conducted under uniform experimental settings to ensure fair and reproducible comparisons. Each algorithm was executed on all 26 benchmark functions with a fixed dimensionality of 30, a maximum of 500 iterations, and a population size of 30. To guarantee statistical reliability, each algorithm was independently run 30 times on each function. Key performance metrics—including the best, mean, and standard deviation of the obtained objective values, as well as computational time—were recorded. Furthermore, the convergence behavior of each algorithm across all test functions was documented. Statistical analyses, including Friedman ranking scores and Wilcoxon signed-rank tests, were performed to rigorously assess and compare the relative performance of the algorithms. These procedures collectively ensure that the experimental evaluation is thorough, systematic, and consistent with established best practices in the literature.

To ensure that the performance evaluation of the proposed algorithm is both comprehensive and representative, this study follows the problem landscape characteristic coverage principle in selecting the benchmark test functions. Specifically, the test set is designed to cover various typical characteristics of optimization problems, thereby enabling a thorough assessment of the algorithm’s capabilities under different difficulty levels and structural conditions. The selected functions fall into the following categories:

- **Unimodal Functions**—These functions have a single global optimum and are primarily used to evaluate the convergence speed and local exploitation ability of the algorithm.
  Examples from the selected set: F1 (Sphere), F2 (Schwefel 2.22), F3 (Schwefel 1.2), F4 (Schwefel 2.21), F5 (Step), F6 (Quartic), F7 (Exponential), F8 (Sum Power), F9 (Sum Square), F10 (Rosenbrock), F11 (Zakharov), F12 (Trid), F13 (Elliptic), and F14 (Cigar).
- **Multimodal Functions**—These functions have multiple local optima, allowing for the assessment of the algorithm’s global search capability and its ability to escape from local optima.
  Examples from the selected set: F15 (Rastrigin), F16 (NCRastrigin), F17 (Ackley), F18 (Griewank), F19 (Alpine), F20 (Penalized 1), F21 (Penalized 2), F23 (Lévy), F24 (Weierstrass), F25 (Solomon), and F26 (Bohachevsky).
- **Separable and Non-separable Functions**—These functions are used to test the algorithm’s adaptability in handling problems with either independent variables or strong inter-variable coupling.
  Examples from the selected set:
  · Separable: F1, F2, F3, F5, F8, F9, and F16.
  · Non-separable: F10, F12, F17, F18, F20, F23, and F24.
- **Ill-conditioned and Anisotropic Functions**—These functions exhibit large variations in gradient magnitude across different search directions, testing the algorithm’s stability in highly non-uniform search spaces.
  Examples from the selected set: F10 (Rosenbrock), F13 (Elliptic), and F14 (Cigar).
- **Non-differentiable/Discontinuous Functions**—These functions are used to evaluate the robustness of the algorithm under conditions where gradient information is unavailable or the function is non-smooth.
  Examples from the selected set: F5 (Step) and F16 (NCRastrigin).
- **Scalable Functions**—These functions allow the dimensionality to be adjusted, enabling the analysis of the algorithm’s computational efficiency and performance trends in high-dimensional spaces.

Examples from the selected set: F1, F2, F3, F4, F7, F8, F9, F10, F11, F12, F13, F15, F16, F17, F18, F20, and F24.

By adopting such a balanced and diverse test set design, the proposed algorithm can be thoroughly evaluated across a variety of search scenarios. This ensures a comprehensive examination of its global search capability, local exploitation ability, convergence speed, and stability, thereby enhancing the generality and persuasiveness of the conclusions.

The selection of these 26 test functions enables a comprehensive and systematic validation of the proposed algorithm’s adaptability and superiority across various types of optimization problems. The detailed information about the test functions can be found in Table 2.

#### 4.1.1. Performance Indicators

To thoroughly assess the performance of the proposed algorithm, 30 independent repetitions of experiments were conducted for the AGG-GCRA and 11 comparison algorithms. The results were analyzed using three metrics: best, mean, and standard deviation (Std). The best value represents the optimal solution achieved across multiple runs, the mean value reflects the overall search performance, and the standard deviation measures the stability of the results. These three metrics enable a thorough evaluation of the algorithm’s convergence accuracy, stability, and global search capacity.

The formulas for calculating the best value, mean, and standard deviation are given in Equations (18)–(20), respectively:

(18) $$Best=min\{f_{1},f_{2},\ldots ,f_{N}\},$$

(19) $$Mean=\frac{1}{N}\sum_{i=1}^{N}\;f_{i},$$

(20) $$Std=\sqrt{\frac{1}{N-1}\sum_{i=1}^{N}\;(f_{i}-Mean{)}^{2}},$$

where $f_{i}$ represents the fitness score obtained from the i-th independent run, and N is the total number of runs, which is 30 in this case.

#### 4.1.2. Numerical Results Analysis

In this study, we conducted a comprehensive evaluation of the proposed AGG-GCRA and 11 comparison algorithms on 26 benchmark functions. Each test was assessed based on five performance metrics: best fitness, average fitness, standard deviation, average computational time, and average ranking based on average fitness. The experimental results are shown in Table 3. Additionally, we included two benchmark algorithms for comparison: Latin Hypercube Sampling combined with parallel local search (LHS-PLS), which generates uniformly distributed initial samples and performs independent local searches from each to enhance global coverage, and pattern search, a classical derivative-free method that explores the neighborhood of the current point along predefined directions and adapts the step size to converge to local optima. Both are typical standard methods for derivative-free optimization. Given that double-precision floating-point calculations typically provide about 16 significant digits of precision, and due to rounding errors, differences below 1 × 10^−8^ are considered insignificant in the analysis. Therefore, all values below this threshold were rounded to zero before analysis.

The numerical results indicate that the AGG-GCRA demonstrates superior optimization performance on most test functions. Specifically, it achieved the best fitness value on 25 out of the 26 benchmark functions, ranking first among all 14 comparison algorithms. The only exception was function F13, where it ranked 12th. The average ranking across all benchmark functions was 1.42, with an overall ranking of first. This suggests that the algorithm exhibits strong and stable performance across a variety of optimization problems.

Furthermore, the standard deviation results confirm the algorithm’s excellent stability: For many functions, the standard deviation was zero, indicating that the AGG-GCRA consistently converged to the same optimal value across all 30 independent runs. The computational time remained within a reasonable range for all test functions, highlighting the practicality of the AGG-GCRA in optimization scenarios.

#### 4.1.3. Convergence Curve Analysis

**Figure 5:**
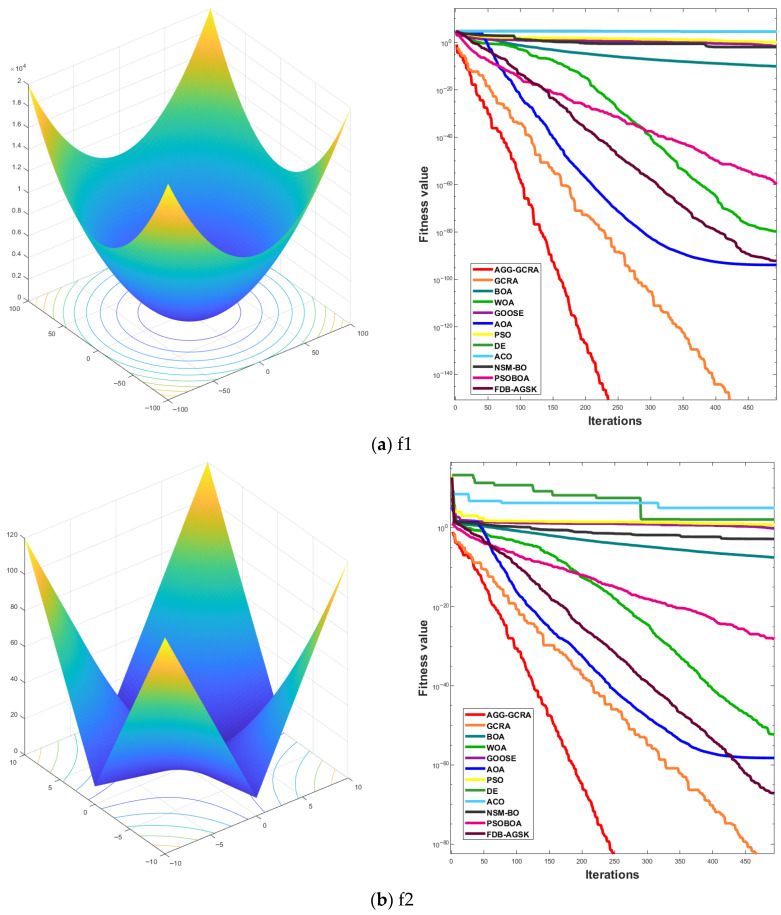

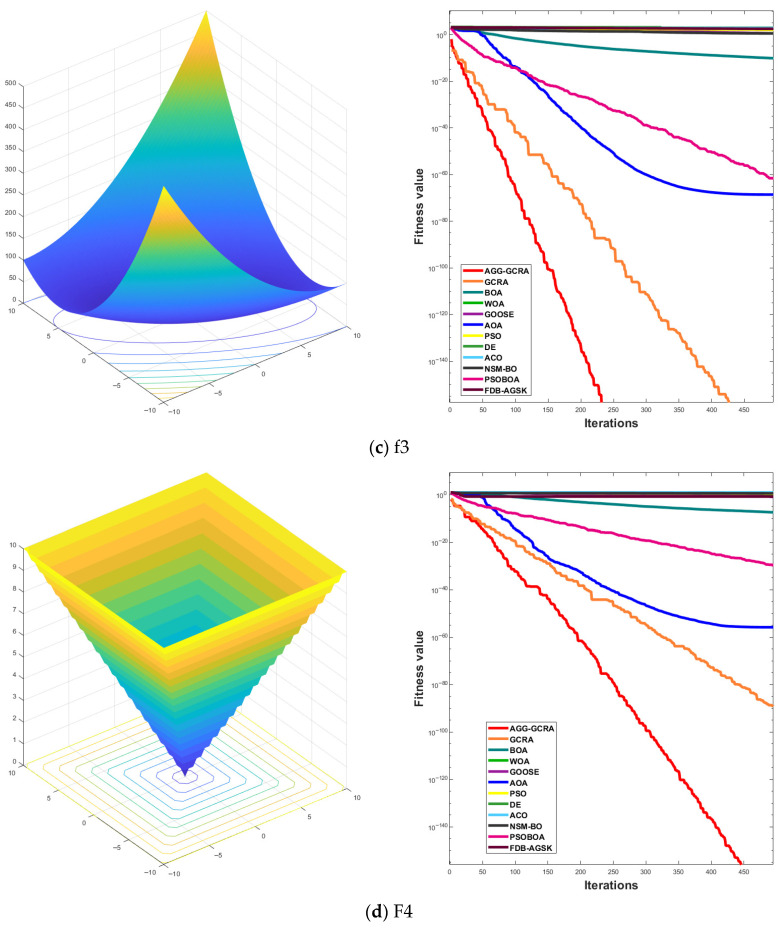

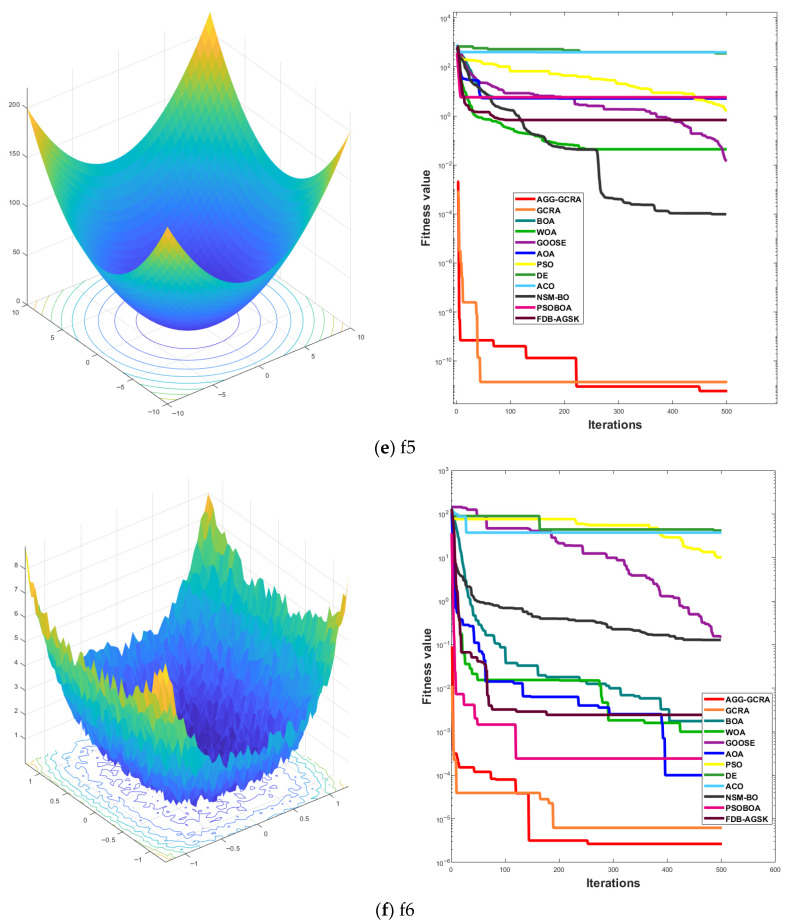

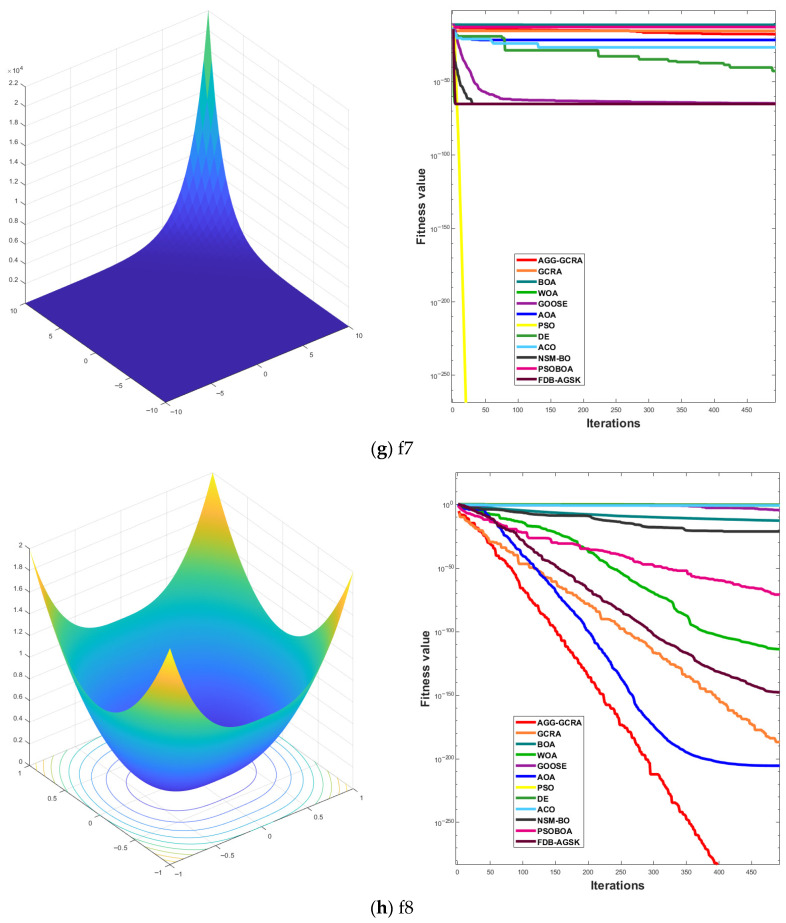

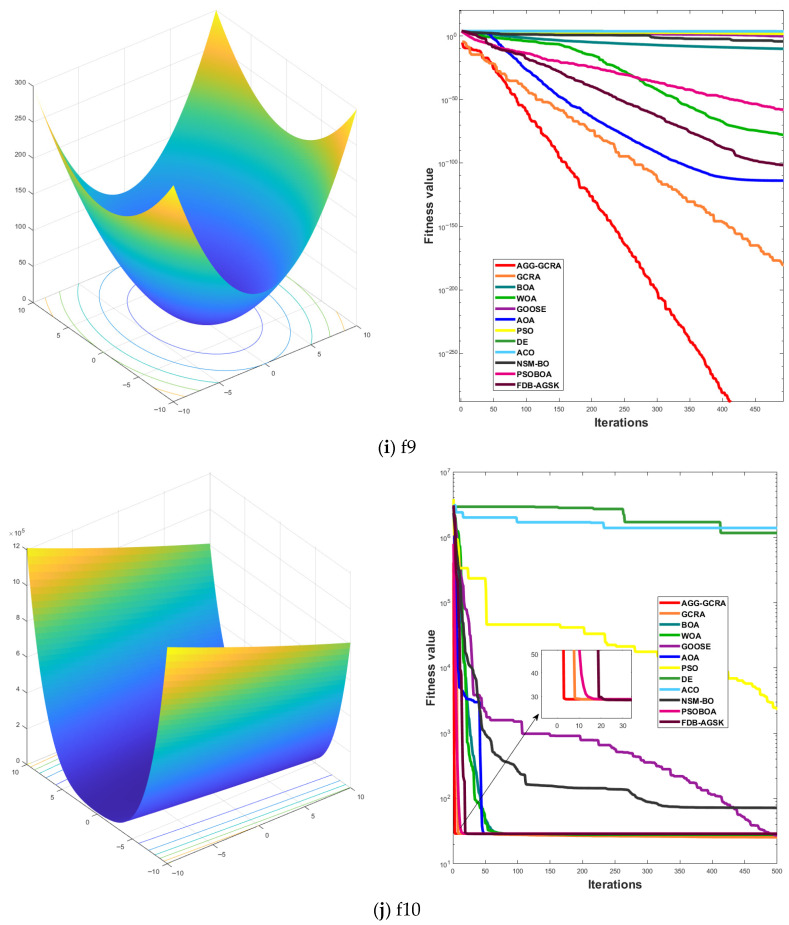

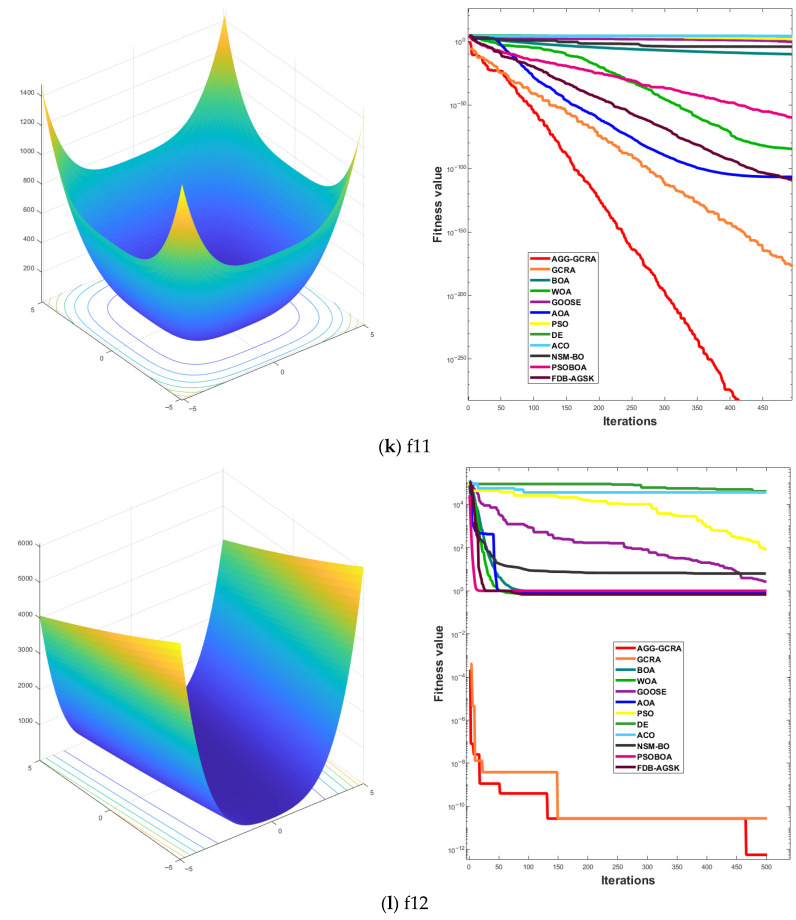

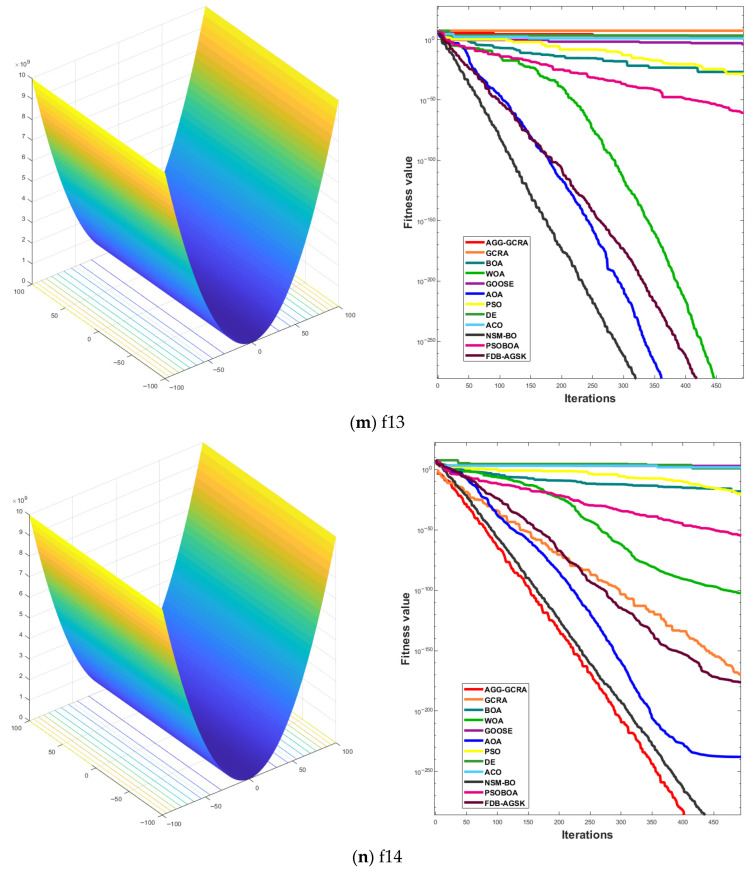

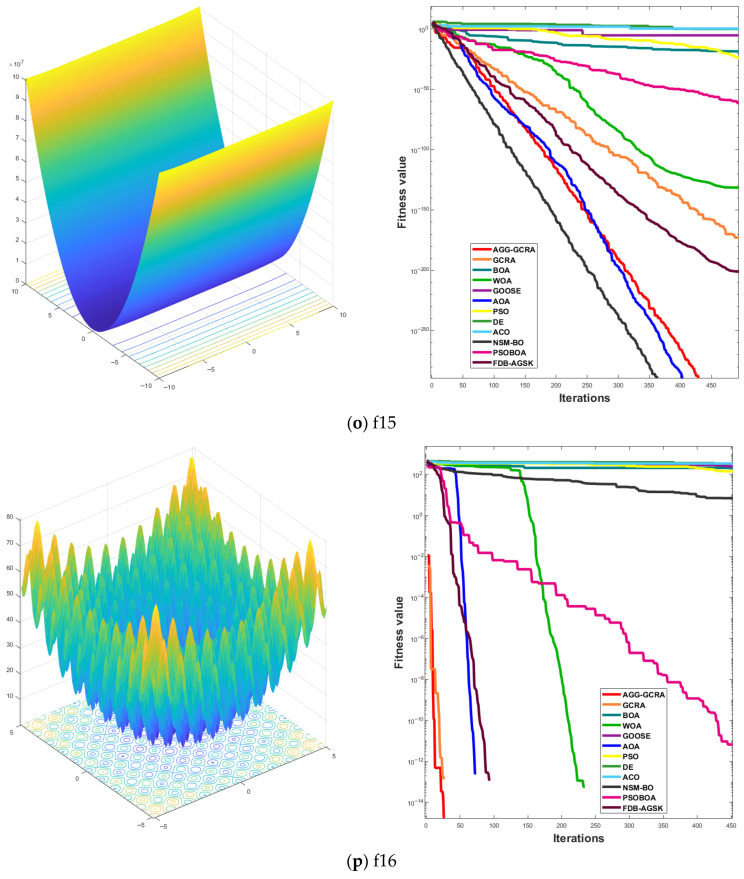

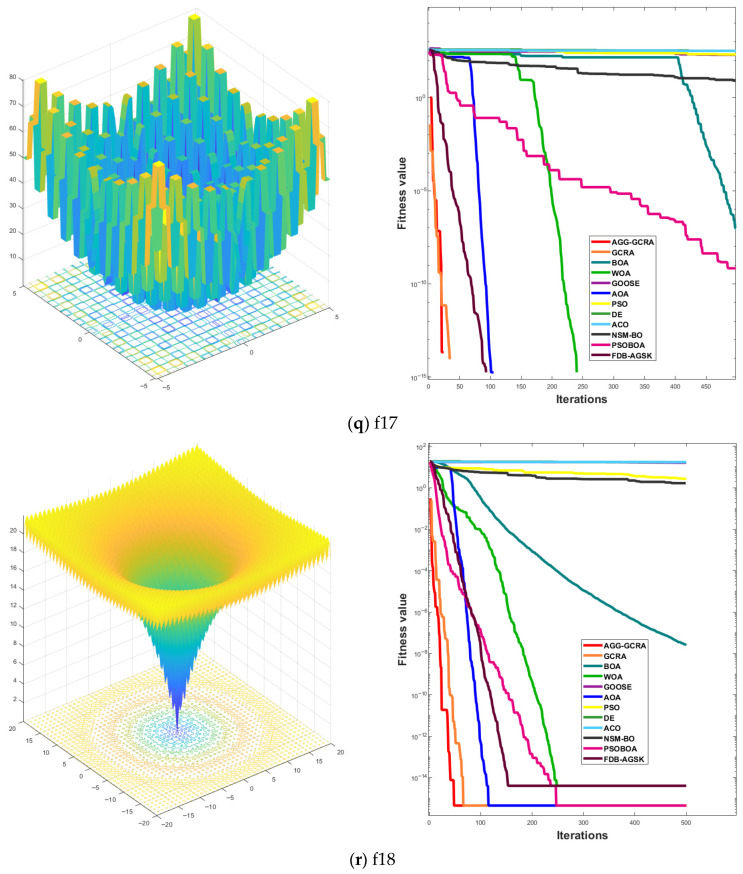

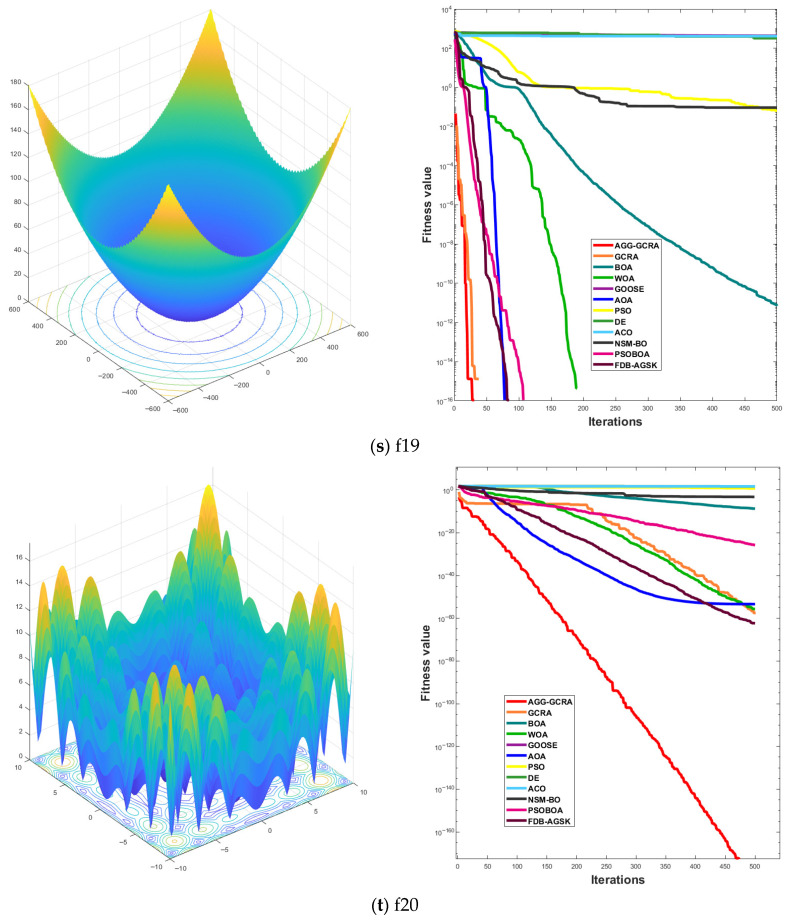

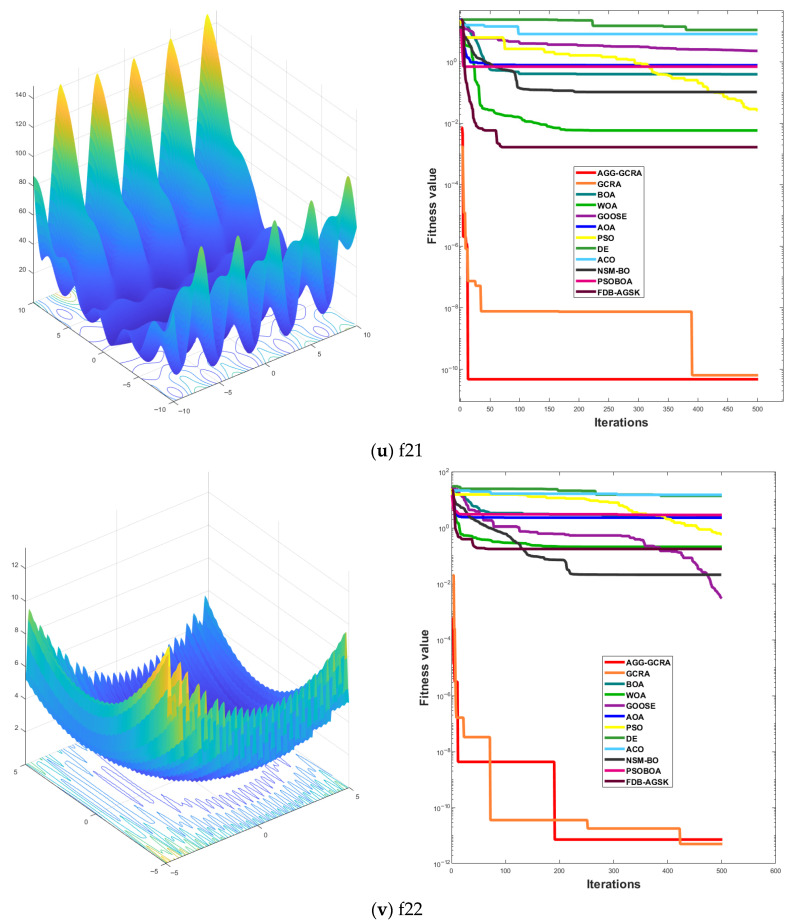

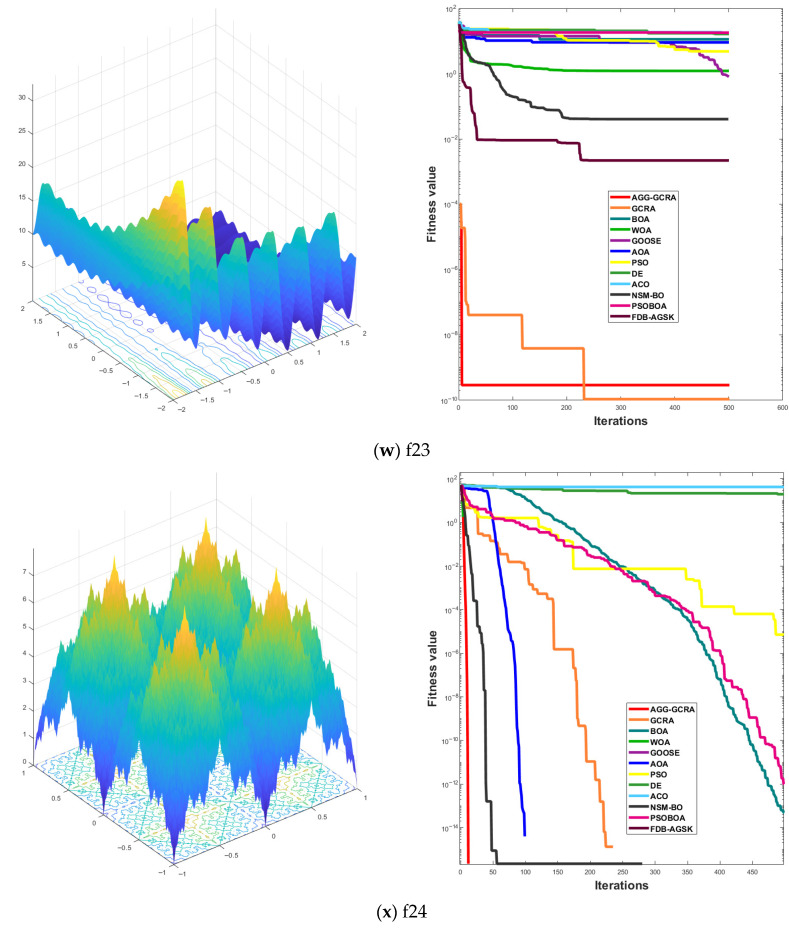

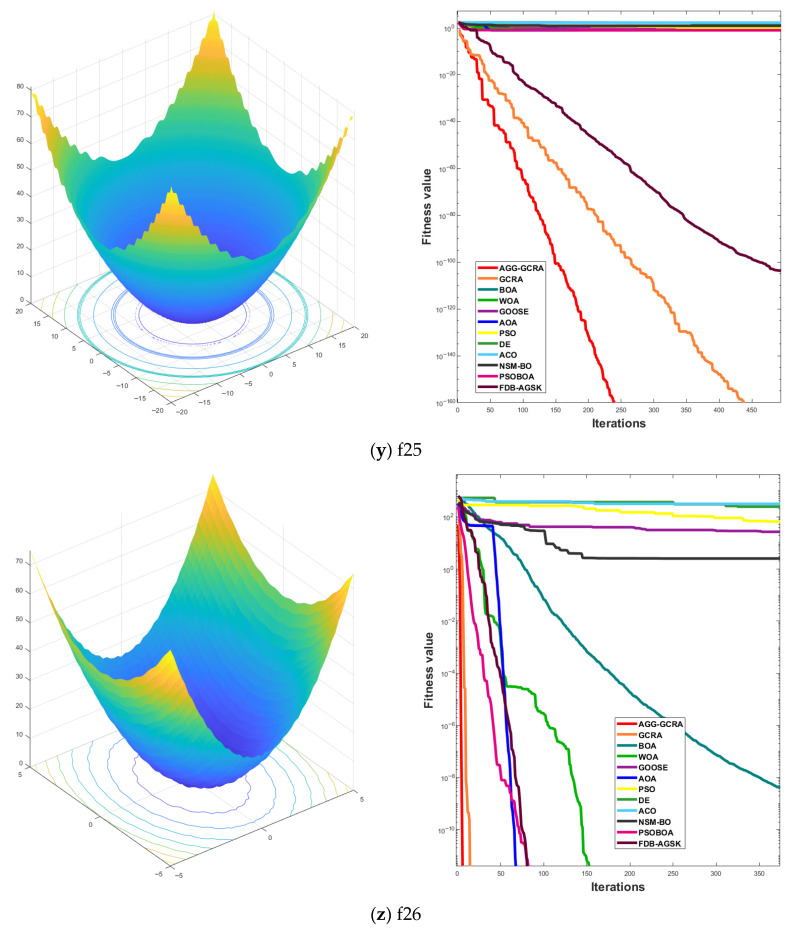
Convergence curves of 12 algorithms across 26 test functions.

To further assess the effectiveness of the proposed AGG-GCRA on standard test functions, this paper examines its convergence curves across 26 test functions in comparison with 11 other algorithms. The experimental results show that the AGG-GCRA is able to approach the optimal solution in fewer iterations on 23 of the test functions (F1–F6, F8–F12, F14, and F16–F26) and demonstrates outstanding stability, showing faster convergence and higher accuracy. This result clearly demonstrates that the AGG-GCRA has a significant advantage in balancing global search and local exploitation capabilities, effectively avoiding local optima and enhancing both optimization efficiency and solution accuracy. The relevant convergence curves are shown in Figure 5.

#### 4.1.4. Friedman Ranking Scores and Wilcoxon Signed-Rank Analysis Results

Based on the results from 26 benchmark test functions, Table 4 presents the Friedman ranking scores and corresponding rankings for the proposed AGG-GCRA; 11 competing algorithms (GCRA, BOA, WOA, GOOSE, AOA, PSO, DE, ACO, NSM-BO, PSOBOA, and FDB-AGSK); and 2 baseline algorithms (LHS combined with parallel local search and Patternsearch).

The AGG-GCRA achieves the lowest Friedman score of 1.7308, ranking first among all 14 algorithms, which demonstrates its superior overall optimization performance. The next best performers are the GCRA (3.3077) and FDB-AGSK (4.9231), indicating that the improvements in the AGG-GCRA provide a noticeable performance gain over its base version. In contrast, the ACO algorithm records the highest score (13.0769) and ranks last, reflecting its relatively weaker performance on the test set. Algorithms such as the BOA, PSO, and GOOSE are positioned in the middle-to-lower ranking range and do not outperform the top-ranked methods. Overall, the Friedman ranking analysis confirms that the AGG-GCRA consistently delivers better performance and stability compared to all other tested algorithms.

At a significance level of α = 0.05, the Wilcoxon signed-rank test was performed to evaluate pairwise performance differences between the AGG-GCRA and each of the other 13 algorithms across the 26 functions. As shown in Table 5, all *p*-values are less than 0.05, indicating statistically significant performance differences in every comparison. Notably, the AGG-GCRA achieves extremely small *p*-values against DE (9.3386 × 10^−6^), ACO (9.3386 × 10^−6^), PSO (7.0443 × 10^−5^), BOA (9.6755 × 10^−5^), and GOOSE (7.0443 × 10^−5^), highlighting its substantial advantage. Even in comparisons with strong competitors such as the GCRA (1.3183 × 10^−4^) and FDB-AGSK (2.3556 × 10^−3^), the AGG-GCRA still shows a statistically significant improvement.

These results, validated by both the Friedman ranking and the Wilcoxon signed-rank test, demonstrate that the AGG-GCRA not only outperforms twelve state-of-the-art algorithms but also surpasses the two baseline optimization strategies in terms of convergence stability, search capability, and robustness.

### 4.2. Application on Six Practical Engineering Problems

To verify the effectiveness of the proposed AGG-GCRA in real-world engineering optimization problems, this paper selects four classic engineering design problems as benchmark test cases and compares the results with 11 mainstream metaheuristic algorithms. All comparison algorithms are executed under identical experimental conditions, with a maximum of 500 iterations and a population size of 30. To ensure the reliability and statistical significance of the results, each set of experiments is independently repeated 20 times.

To comprehensively evaluate the performance of each algorithm on engineering optimization problems, this paper employs several evaluation metrics, including the best value, mean value, standard deviation, median, and average computational time (ACT). The best value represents the minimum objective function value obtained from 20 independent runs, reflecting the algorithm’s maximum optimization capability in a single run. The mean value is calculated by averaging the objective function values across 20 experiments, reflecting the algorithm’s overall performance. The standard deviation measures the fluctuation range of the 20 results, reflecting the stability and robustness of the algorithm. The median is the middle value of the 20 results, further revealing the distribution characteristics and minimizing the impact of outliers. ACT indicate the average time (in seconds) the algorithm takes to finish each optimization task over several runs, serving as a measure of its computational efficiency.

#### 4.2.1. Weight Minimization of a Speed Reducer

**Figure 6:**
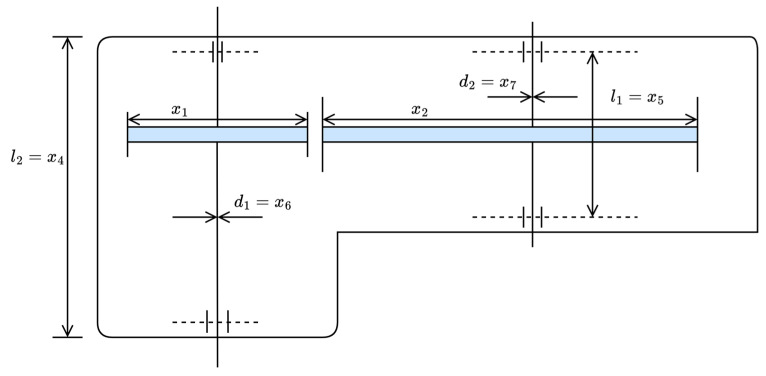
Schematic diagram of weight minimization of a speed reducer problem.

Figure 6 illustrates the schematic diagram of the weight minimization of a speed reducer problem [60]. This problem focuses on minimizing the weight of the speed reducer structure while satisfying engineering constraints related to gear meshing, bearing strength, bending stress, and dimensions. It involves complex nonlinear relationships and multiple constraints, making it a classic example of mechanical structural optimization, ideal for evaluating the performance of optimization algorithms when applied to real-world engineering problems.

The problem involves optimizing seven parameters: shaft 1 diameter $d_{1}$, shaft 2 diameter $d_{2}$, shaft 1 length between bearings $l_{1}$, shaft 2 length between bearings $l_{2}$, number of pinion teeth z, teeth module m, and face width b. The formula for calculating the minimum weight is shown in Equation (21):

(21) $$\begin{array}{l} \min\;f(x)=0.7854x_{1}x_{2}^{2}(3.3333x_{3}^{2}-43.0934+14.9334x_{3})\\ \;\;\;\;-1.508x_{1}(x_{6}^{2}+x_{7}^{2})+7.4777(x_{6}^{3}+x_{7}^{3})+0.7854(x_{4}x_{6}^{2}+x_{5}x_{7}^{2}) \end{array}$$

where $x_{1}=b$, $x_{2}=m$, $x_{3}=z,x_{4}=l_{1}$, $x_{5}=l_{2}$, $x_{6}=d_{1}$, and $x_{7}=d_{2}$, subject to:

$$g_{1}(x)=\frac{27}{x_{1}x_{2}x_{3}}-1\leq 0,$$

$$g_{2}(x)=\frac{397.5}{x_{1}x_{2}^{2}x_{3}}-1\leq 0,$$

$$g_{3}(x)=\frac{1.93x_{4}^{3}}{x_{2}x_{3}x_{6}^{4}}-1\leq 0,$$

$$g_{4}(x)=\frac{1.93x_{5}^{3}}{x_{2}x_{3}x_{7}^{4}}-1\leq 0,$$

$$g_{5}(x)=\sqrt{{\left(\frac{745x_{4}}{x_{2}x_{3}}\right)}^{2}+\frac{16.9\times 10^{6}}{110x_{6}^{3}}}-1\leq 0,$$

$$g_{6}(x)=\sqrt{{\left(\frac{745x_{5}}{x_{2}x_{3}}\right)}^{2}+\frac{157.5\times 10^{6}}{85x_{7}^{3}}}-1\leq 0,$$

$$g_{7}(x)=\frac{x_{2}x_{3}}{40}-1\leq 0,$$

$$g_{8}(x)=\frac{5x_{2}}{x_{1}}-1\leq 0,$$

$$g_{9}(x)=\frac{x_{1}}{12x_{2}}-1\leq 0,$$

$$g_{10}(x)=\frac{1.56x_{6}+1.9}{x_{4}}-1\leq 0,$$

$$g_{11}(x)=\frac{1.1x_{7}+1.9}{x_{5}}-1\leq 0,$$

$$2.6\leq x_{1}\leq 3.6,$$

$$0.7\leq x_{2}\leq 0.8,$$

$$17\leq x_{3}\leq 28,$$

$$7.3\leq x_{4}\leq 8.3,$$

$$7.8\leq x_{5}\leq 8.3,$$

$$2.9\leq x_{6}\leq 3.9,$$

$$5\leq x_{7}\leq 5.5$$

In this problem, we present the optimal values and corresponding parameter configurations obtained by each algorithm during the runs (Table 6), along with statistical metrics such as best value, mean value, median, and standard deviation (Table 7). The algorithms are ranked based on the mean value. Based on the experimental results, it is clear that the AGG-GCRA achieved the optimal value of 2994.2343 and the ACTs remain reasonable. Overall, the AGG-GCRA significantly outperforms most of the comparison algorithms.

#### 4.2.2. Tension/Compression Spring Design Problem

**Figure 7:**
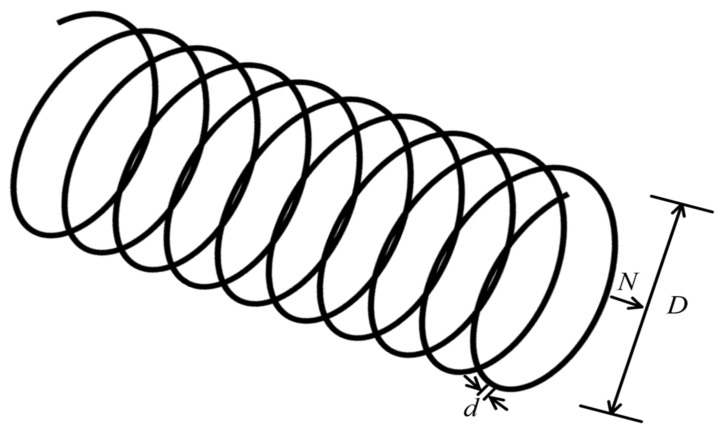
Schematic diagram of the spring design problem.

Figure 7 illustrates the schematic diagram of the tension/compression spring design problem [61]. The objective of this problem is to minimize the spring weight while meeting constraints related to shear stress, deflection, natural frequency, and geometric dimensions. With its solid engineering background and clear constraint system, this problem is widely used to evaluate the adaptability and convergence performance of optimization algorithms in structural optimization.

The design problem includes three parameters to be optimized: the wire diameter (d), the mean coil diameter (D), and the number of active coils (N). The formula for calculating the minimum weight is shown in Equation (22):

(22) $$minf(x)=(x_{3}+2)x_{2}x_{1}^{2}$$

where $x_{1}=d$, $x_{2}=D$, and $x_{3}=N$, subject to:

$$g_{1}(x)=1-\frac{x_{2}^{3}x_{3}}{71,785x_{1}^{4}}\leq 0,$$

$$g_{2}(x)=\frac{4x_{2}^{2}-x_{1}x_{2}}{12,566(x_{2}x_{1}^{3}-x_{1}^{4})}+\frac{1}{5108x_{1}^{2}}-1\leq 0,$$

$$g_{3}(x)=1-\frac{140.45x_{1}}{x_{2}x_{3}}\leq 0,$$

$$g_{4}(x)=\frac{x_{1}+x_{2}}{1.5}-1\leq 0$$

$$0.5\leq x_{1},x_{2}\leq 2,2{\leq x}_{3}\leq 15,$$

In this problem, we present the optimal values and corresponding parameter configurations obtained by each algorithm during the runs (Table 8), along with statistical metrics such as best value, mean value, median, and standard deviation (Table 9). The algorithms are ranked based on their mean values. Based on the experimental results, it is clear that the AGG-GCRA achieved the optimal value of 0.0127, with a success rate of 100% across 20 independent runs, and the ACTs remain reasonable. Moreover, its mean value ranks first, indicating the algorithm’s excellent optimization ability and stability. Overall, the AGG-GCRA significantly outperforms most of the comparison algorithms.

#### 4.2.3. Welded Beam Design Problem

**Figure 8:**
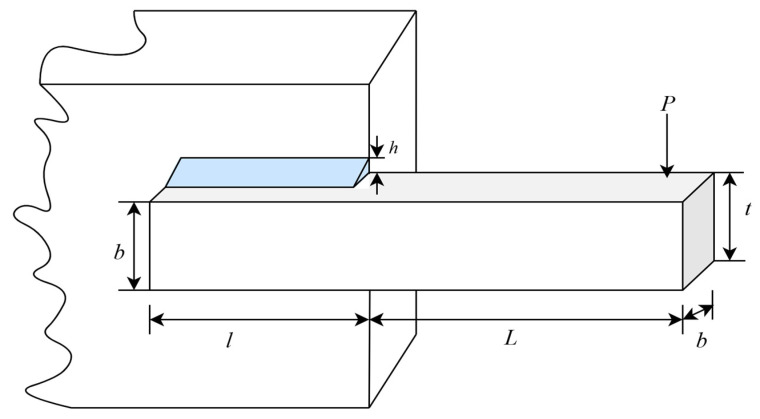
Schematic diagram of the welded beam design problem.

Figure 8 illustrates the schematic diagram of the welded beam design problem [62]. The goal is to minimize the manufacturing cost of the welded beam structure while considering engineering constraints related to strength, deflection, and stability. This problem involves balancing material costs and mechanical performance, with nonlinear and multi-constraint characteristics, making it ideal for evaluating the ability of optimization algorithms to handle multi-objective trade-offs and constraint management.

The welded beam design problem involves four design parameters: weld thickness (h), welding rod length (l), rod height (t), and rod thickness (b). The formula for minimizing manufacturing costs is shown in Equation (23):

(23) $$minf(x)=1.10471x_{1}^{2}x_{2}+0.04811x_{3}x_{4}(14+x_{2})$$

where $x_{1}=h$, $x_{2}=l$, $x_{3}=t$, and $x_{4}=b$, subject to:

$$g_{1}(x)=\tau (x)+\tau_{max}\leq 0,$$

$$g_{2}(x)=\sigma (x)+\sigma_{max}\leq 0,$$

$$g_{3}(x)=\delta (x)+\delta_{max}\leq 0,$$

$$g_{4}(x)=x_{1}-x_{4}\leq 0,$$

$$g_{5}(x)=P-P_{c}(x)\leq 0,$$

$$g_{6}(x)=0.125-x_{1}\leq 0,$$

$$g_{7}(x)=0.10471x_{1}^{2}+0.04811x_{3}x_{4}(14+x_{2})-5\leq 0,$$

$$0.1\leq x_{1},x_{4}\leq 2.0,0.1\leq x_{2},x_{3}\leq 10.0,$$

where

$$\tau (x)=\sqrt{(\tau^{\prime }{)}^{2}+2\tau^{\prime }\tau^{\prime \prime }\frac{x_{2}^{2}}{2R}+(\tau^{\prime \prime }{)}^{2}},\tau^{\prime }=\frac{P}{\sqrt{2x_{1}x_{2}}},\tau^{\prime \prime }=\frac{MR}{J},$$

$$M=P\left(L+\frac{x_{2}}{2}\right),R=\sqrt{\frac{x_{2}^{2}}{4}+{\left(\frac{x_{1}+x_{3}}{2}\right)}^{2}},J=2\left\{\sqrt{2x_{1}x_{2}}\left[\frac{x_{3}^{2}}{4}+{\left(\frac{x_{1}+x_{3}}{2}\right)}^{2}\right]\right\},$$

$$\sigma \left(x\right)=\frac{6PL}{x_{4}x_{3}^{2}},\delta \left(x\right)=\frac{4PL^{3}}{Ex_{4}x_{3}^{3}},P_{c}\left(x\right)=\frac{4.013E\sqrt{\frac{x_{3}^{2}x_{4}^{6}}{36}}}{L^{2}}\left\{1-\frac{x_{3}}{2L}\sqrt{\frac{E}{4G}}\right\},$$

$$P=6000\;lb,L=14\;in,E=30\times 10^{6}psi,G=12\times 10^{6}psi,\tau_{max}=13,600\;psi,\sigma \left(x\right)=30,000\;psi,\delta \left(x\right)=0.25in.$$

In this problem, we present the optimal values and corresponding parameter configurations obtained by each algorithm during the runs (Table 10), along with statistical metrics such as best value, mean value, median, and standard deviation (Table 11). The algorithms are ranked according to their mean value. Based on the experimental results, it is clear that the AGG-GCRA achieved the optimal value of 1.6702, with a success rate of 90% across 20 independent runs, and the ACTs remain reasonable. Moreover, its mean value ranks first, indicating the algorithm’s excellent optimization ability and stability. Overall, the AGG-GCRA significantly outperforms most of the comparison algorithms.

#### 4.2.4. Gas Transmission Compressor Design (GTCD) Problem

**Figure 9:**
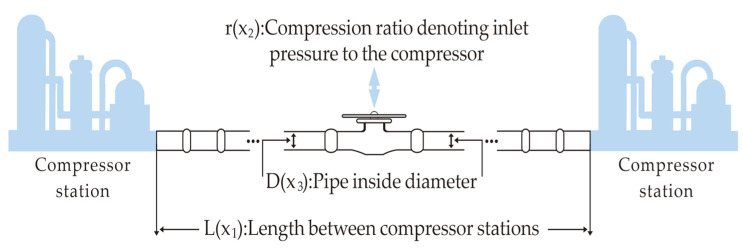
Schematic diagram of the GTCD problem.

Figure 9 illustrates the schematic diagram of the GTCD problem [63]. This problem focuses on minimizing the cost of the natural gas compressor system, with constraints related to fluid dynamics principles and engineering safety requirements. This problem demonstrates notable nonlinear characteristics and practical engineering significance, making it a standard test case for assessing the performance of optimization algorithms in the design of energy and process systems.

In this problem, the objective is to determine the optimal values of three parameters, L, r, and D, to minimize the value of C_1_. Specifically, L represents the distance between two compressor stations; r denotes the compression ratio at the compressor inlet, calculated as P_1_ divided by P_2_, where P_1_ is the outlet pressure and P_2_ is the inlet pressure (both measured in psi); and D refers to the pipeline’s internal diameter (in inches). The formula for calculating the minimum weight is shown in Equation (24):

(24) $$\begin{array}{l} minf(x)=8.61\times 10^{5}{{{x_{1}}^{\frac{1}{2}}x_{2}x}_{3}}^{-\frac{2}{3}}{\left({x_{2}}^{2}-1\right)}^{-\frac{1}{2}}\\ \;\;\;\;-7.6543\times 10^{8}{x_{1}}^{-1}+3.69\times 10^{4}x_{3}\\ \;\;\;\;+7.72\times 10^{8}{x_{1}}^{-1}{x_{2}}^{0.219}, \end{array}$$

where $x_{1}=L$, $x_{2}=r$, and $x_{3}=D$, subject to:

$$10\leq x_{1}\leq 55,$$

$$1.1\leq x_{2}\leq 2,$$

$$10\leq x_{3}\leq 40.$$

In this problem, we report the optimal values and corresponding parameter configurations achieved by each algorithm during the runs (Table 12), as well as statistical metrics such as best value, mean value, median, and standard deviation (Table 13). The algorithms are ranked based on the mean value. From the experimental results, it is evident that the AGG-GCRA achieved the optimal value of 1,677,759.276 and the ACTs remain reasonable. Moreover, its mean value ranks first, with a standard deviation of 0, indicating the algorithm’s excellent optimization ability and stability. Overall, the AGG-GCRA significantly outperforms most of the comparison algorithms.

#### 4.2.5. Three-Bar Truss Design Problem

**Figure 10:**
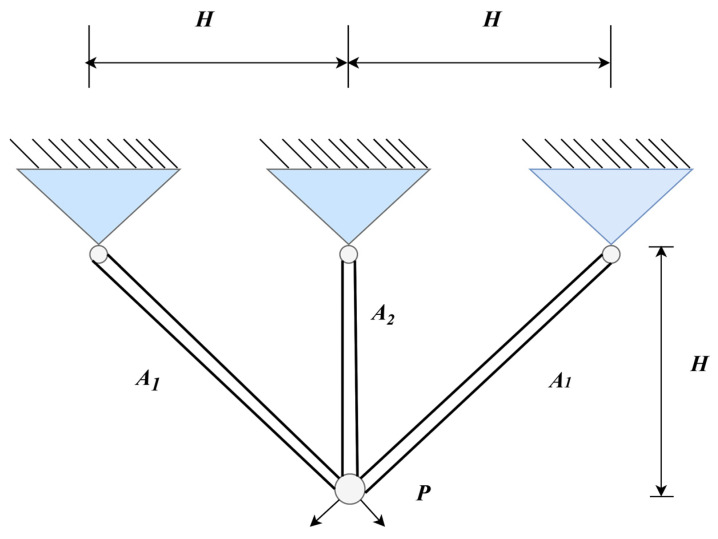
Schematic diagram of the three-bar truss design.

Figure 10 illustrates the schematic diagram of the three-bar truss design problem [64]. The goal is to minimize the total mass of the truss, with design variables generally consisting of the cross-sectional area or dimensions of each bar. These parameters must be properly adjusted within the stress and geometric constraints to optimize the structure. Although the problem is of modest size, its clear structure and well-defined constraints make it an ideal standard test case for evaluating and comparing the basic performance of optimization algorithms.

The formula for calculating the minimum weight is shown in Equation (25):

(25) $$minf(x)=\left(2\sqrt{2}x_{1}+x_{2}\right)\times H$$

where $x_{1}=A_{1}$, $x_{2}=A_{2}$:

$$g_{1}(x)=\frac{\sqrt{2}x_{1}+x_{2}}{\sqrt{2}x_{1}+\frac{2x_{1}x_{2}}{x_{2}}}P-\sigma \leq 0,$$

$$g_{2}(x)=\frac{P}{\sqrt{2}x_{1}+\frac{2x_{1}x_{2}}{x_{2}}}-\sigma \leq 0,$$

$$g_{3}(x)=\frac{P}{x_{1}+\sqrt{2}x_{2}}-\sigma \leq 0,$$

$$0\leq x_{1},x_{2}\leq 1.$$

where $H=1000\;mm,\;P=2{kN/cm}^{2},\sigma =2\;{kN/cm}^{2}$.

In this problem, we report the optimal values and corresponding parameter configurations achieved by each algorithm during the runs (Table 14), as well as statistical metrics such as best value, mean value, median, and standard deviation (Table 15). The algorithms are ranked based on the mean value. From the experimental results, it is evident that the AGG-GCRA achieved the optimal value of 263.8523 and the ACTs remain reasonable. Moreover, its mean value ranks first, with a standard deviation of 0, indicating the algorithm’s excellent optimization ability and stability. Overall, the AGG-GCRA significantly outperforms most of the comparison algorithms.

#### 4.2.6. Multiple-Disk Clutch Brake Design Problem

**Figure 11:**
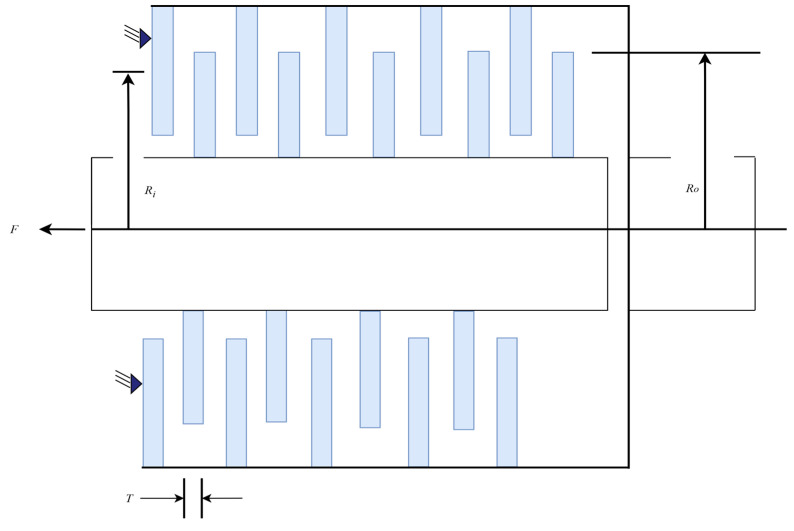
Schematic diagram of the multiple-disk clutch brake design problem.

Figure 11 presents the schematic diagram of the multiple-disk clutch brake design problem [65]. The objective of this problem is to reduce the weight of the clutch/brake system while adhering to constraints such as friction torque, contact pressure, and rotational speed limits. The problem features a practical engineering context and a complex system of physical constraints, making it an important reference case for evaluating the performance of optimization algorithms in high-complexity, multi-constraint environments.

In this problem, five parameters are considered as decision variables: the number of friction surfaces Z and the driving force F, as well as three dimensional parameters—the disk thickness t, outer radius $r_{o}$, and inner radius $r_{i}$—all measured in millimeters. The formula for calculating the minimum weight is shown in Equation (26):

(26) $$minf(x)=\pi (x_{2}^{2}-x_{1}^{2})x_{3}(x_{5}+1)p_{m}$$

where $x_{1}=r_{i}$, $x_{2}=$, $x_{3}=t$, $x_{4}=F$, $x_{5}=Z$, subject to:

$$g_{1}(x)=x_{2}-x_{1}-\Delta R\geq 0$$

$$g_{2}(x)=L_{max}-(Z+1)(t+\delta )\geq 0$$

$$g_{3}(x)=p_{max}-p_{rz}\geq 0$$

$$g_{4}(x)=p_{max}V_{sr,max}-p_{rz}V_{sr}\geq 0$$

$$g_{5}(x)=V_{sr,max}-V_{sr}\geq 0$$

$$g_{6}(x)=M_{h}-sM_{s}\geq 0$$

$$g_{7}(x)=T\geq 0$$

$$g_{8}(x)=T_{max}-T\geq 0$$

$$60\leq x_{1}\leq 80\;mm,90\leq x_{2}\leq 110\;mm,1.5\leq x_{3}\leq 3\;mm,0\leq x_{4}\leq 1000\;N,2\leq x_{5}\leq 9$$

where:

$$p_{m}=0.0000078\;{kg/mm}^{3},p_{max}=1\;MPa,\mu =0.5,V_{sr,max}=10\;m/s,s=1.5,T_{max}=15\;s,$$

$$n=250\;rpm,M_{f}=3\;Nm,I_{z}=55\;{kg/m}^{2},\delta =0.5\;mm,\Delta R=20\;mm,L_{max}=30\;mm,$$

$$M_{h}=\frac{2}{3}\mu x_{4}x_{5}\frac{x_{2}^{3}-x_{1}^{3}}{x_{2}^{2}-x_{1}^{2}}N\;mm,w=\frac{\pi n}{30}\frac{rad}{s},R_{sr}=\frac{2}{3}\frac{x_{2}^{3}-x_{1}^{3}}{x_{2}^{2}-x_{1}^{2}}mm,A=\pi (x_{2}^{2}-x_{1}^{2}){mm}^{2},$$

$$M_{s}=40\;Nm,p_{rz}=\frac{x_{4}}{A}{N/mm}^{2},V_{sr}=\frac{\pi R_{sr}n}{30}mm/s$$

In this problem, we report the optimal values and corresponding parameter configurations achieved by each algorithm during the runs (Table 16), as well as statistical metrics such as best value, mean value, median, and standard deviation (Table 17). The algorithms are ranked based on the mean value. From the experimental results, it is evident that the AGG-GCRA achieved the optimal value of 0.2352 and the ACTs remain reasonable. Moreover, its mean value ranks first, with a standard deviation of 0, indicating the algorithm’s excellent optimization ability and stability. Overall, the AGG-GCRA significantly outperforms most of the comparison algorithms.

## 5. Discussion

The superiority of the proposed AGG-GCRA stems from several critical improvements. The introduction of a global optimum guidance term effectively steers search agents toward promising areas, reducing the risk of premature convergence. Meanwhile, adaptive parameter adjustment dynamically balances exploration and exploitation, enhancing both search efficiency and convergence speed. Moreover, the elite preservation mechanism retains high-quality solutions throughout the iterations, improving robustness. Coupled with a local perturbation strategy that blends Gaussian noise and Lévy flights, the algorithm maintains population diversity and performs both fine local searches and occasional large jumps to escape local optima, which boosts performance on complex multimodal problems.

From simulation results, the animal-inspired strategies in the GCRA and AGG-GCRA reveal valuable insights from real-world behavior. The greater cane rat’s alternating foraging strategy naturally balances exploration and exploitation. Our experiments show that mimicking this adaptive switching improves escaping local optima and convergence to global solutions. This dual-phase behavior, common in various species, may be evolutionarily advantageous for resource acquisition in uncertain environments, and their computational analogs are effective for optimization.

Compared to popular algorithms like PSO, WOA, and the original GCRA, the AGG-GCRA achieves better solution quality, stability, and consistency. The biological inspiration from the cane rat’s strategy offers a novel framework to switch intelligently between search phases, resulting in superior adaptive behavior. The key novelty lies in integrating adaptive global guidance, elite retention, and local perturbation, balancing exploration and exploitation to tackle a broad spectrum of challenging optimization problems.

## 6. Conclusions

This paper presents the AGG-GCRA that improves upon the original GCRA in convergence speed, solution accuracy, and stability by incorporating global optimum guidance, adaptive parameter tuning, elite preservation, and local perturbations. Extensive tests on 26 benchmark functions and six engineering problems show that the AGG-GCRA outperforms most competitors across multiple metrics, demonstrating excellent convergence and robustness.

Notably, the AGG-GCRA achieved zero standard deviation and top ranking in mean values, validating its strong global optimization ability and reproducibility. Furthermore, the AGG-GCRA maintains reasonable computational time, showing promise for practical applications. Overall, the AGG-GCRA is an efficient, stable, and broadly applicable intelligent optimization tool. However, the algorithm’s added mechanisms increase computational overhead, especially for high-dimensional or costly functions. The adaptive parameter tuning adds complexity to configurations, requiring experience or trial runs. Despite improved local perturbation, premature convergence may still occur in very complex landscapes. Also, the current design targets single-objective static problems and lacks extensions to dynamic or multi-objective cases, limiting applicability in some scenarios.

In summary, the AGG-GCRA strikes a balance between enhanced optimization and computational cost, and users should weigh solution quality against resource constraints. Future work will focus on extending the AGG-GCRA to multi-objective and dynamic optimization problems; exploring large-scale and real-world engineering applications; and integrating recent advances in deep learning and meta-learning to further enhance its performance, adaptability, and broader applicability.

## Figures and Tables

**Table 1:** Parameter settings of 12 algorithms.

Algorithm	Parameter	Algorithm	Parameter
ALL	Max iteration = 500, Agents = 30, Runs = 30, dim = 30	PSO	w_max=0.9,w_min=0.2,c1=2,c2=2
AGG-GCRA	$l=1,x=1,y=4,GR\_c=rand,GR\_c=0.8-0.6*(l/T_{max}$)	DE	p=0.5,CR=0.9
GCRA	l=1,x=1,y=4,GR_c=rand	ACO	tau=1,eta=1,alpha=1, beta=0.1,rho=0.2
BOA	$p=0.6,{power}_{exponent}=0.1$ sensory_modality=0.01	NSM-BO	$p_{{xgm}_{initial}}=0.03;scab=1.25,scsb=1.3,$ rcpp=0.0035,tsgs_factor_max=0.05
WOA	a=linear decrease from 2 to 0, a2=linear decrease from −1 to −2 C=random [0,2]	PSOBOA	$p=0.6,{power}_{exponent}=0.1,$ senso−ry_modality=0.01
GOOSE	$S_{W_{min}}=5,S_{W_{max}}=25,\;coe\_min=0.17$	FDB-AGSK	KF_pool=[0.1,1.0,0.5,1.0], KR_pool=[0.2,0.1,0.9,0.9]
AOA	C1=2,C2=6,C3=1,C4=2,u=0.9, l=0.1		

**Table 2:** Details of the 26 test functions.

s/n	Category	Function Name	Formula	$f_{min}^{*}$	Range
F1	Unimodal	Sphere	$f_{1}(x)=\sum_{i=1}^{dim}\;x_{i}^{2}$	0	[−100, 100]
F2	Unimodal	Schwefel 2.22	$f_{2}\left(x\right)=\prod_{i=1}^{dim}\;\left|x_{i}\right|+\sum_{i=1}^{dim}\;|x_{i}|$	0	[−10, 10]
F3	Unimodal	Schwefel 1.2	$f_{3}(x)=\sum_{i=1}^{dim}\;{\left(\sum_{j=1}^{i}\;x_{j}\right)}^{2}$	0	[−100, 100]
F4	Unimodal	Schwefel 2.21	$f_{4}(x)=\underset{i}{max}\;\{|x_{i}|\},1\leq i\leq dim$	0	[−100, 100]
F5	Unimodal	Step	$f_{5}(x)=\sum_{i=1}^{dim}\;{\left(0.5+x_{i}\right)}^{2}$	0	[−100, 100]
F6	Unimodal	Quartic	$f_{6}(x)=\sum_{i=1}^{dim}\;ix_{i}^{4}+rand$	0	[−1.28, 1.28]
F7	Unimodal	Exponential	$f_{7}(x)=\sum_{i=1}^{dim}\;(e^{x_{i}}-x_{i})$	0	[−10, 10]
F8	Unimodal	Sum power	$f_{8}(x)=\sum_{i=1}^{dim}\;{\left|x_{i}\right|}^{i+1}$	0	[−1, 1]
F9	Unimodal	Sum square	$f_{9}(x)=\sum_{i=1}^{dim}\;ix_{i}^{2}$	0	[−10, 10]
F10	Unimodal	Rosenbrock	$f_{10}(x)=\sum_{i=1}^{dim-1}\;\left((x_{i}-1{)}^{2}+100(x_{i+1}-x_{i}^{2}{)}^{2}\right)$	0	[−5, 10]
F11	Unimodal	Zakharov	$f_{11}(x)={\left(\sum_{i=1}^{dim}\;0.5ix_{i}\right)}^{2}+\sum_{i=1}^{dim}\;x_{i}^{2}+{\left(\sum_{i=1}^{dim}\;0.5ix_{i}\right)}^{4}$	0	[−5, 10]
F12	Unimodal	Trid	$f_{12}(x)=\sum_{i=1}^{dim}\;(1-x_{i}{)}^{2}-\sum_{i=2}^{dim}\;x_{i}x_{i-1}$	0	[−5, 10]
F13	Unimodal	Elliptic	$f_{13}(x)=\sum_{i=1}^{dim}\;(10^{6}{)}^{i/(dim-1)}x_{i}^{2}$	0	[−100, 100]
F14	Unimodal	Cigar	$f_{14}(x)=10^{6}\sum_{i=2}^{dim}\;x_{i}^{2}+x_{1}^{2}$	0	[−100, 100]
F15	Fixed	Rastrigin	$f_{15}(x)=\sum_{i=1}^{dim}\;\left(10-10cos(2\pi x_{i})+x_{i}^{2}\right)$	0	[−5.12, 5.12]
F16	Multimodal	NCRastrigin	$f_{16}(x)=\sum_{i=1}^{dim}\;\left(10-10cos(2\pi x_{i})+x_{i}^{2}\right),x_{i}=\left\{\begin{array}{ll} x_{i}, & \mathrm{if}x_{i}\leq 0.5\\ x_{i}-1, & \mathrm{otherwise} \end{array}\right.$	0	[−5.12, 5.12]
F17	Multimodal	Ackley	$f_{17}(x)=e^{-1}\sum_{i=1}^{dim}\;cos(2\pi x_{i})+20e^{-0.2\sqrt{\frac{1}{dim}\sum_{i=1}^{dim}\;x_{i}^{2}}}+20+e$	0	[−50, 50]
F18	Multimodal	Griewank	$f_{18}(x)=1-\prod_{i=1}^{dim}\;cos\left(\frac{x_{i}}{\sqrt{i}}\right)+\frac{1}{4000}\sum_{i=1}^{dim}\;x_{i}^{2}$	0	[−600, 600]
F19	Fixed	Alpine	$f_{19}(x)=\sum_{i=1}^{dim}\;|{0.1x_{i}+x}_{i}sin(x_{i})|$	0	[−10, 10]
F20	Multimodal	Penalized 1	$f_{20}\left(x\right)=\frac{\pi }{dim}\left\{\sum_{i=1}^{dim-1}\;(y_{i}-1{)}^{2}\left[1+10\sin^{2}\left(\pi y_{i+1}\right)\right]+10{sin}^{2}(\pi y_{1})\right.\left.+(y_{dim}-1{)}^{2}\right\}+\sum_{i=1}^{dim}\;u\left(x_{i},10,100,4\right),y_{i}=1+\frac{x_{i}+1}{4},u(x_{i},a,k,m)=\left\{\begin{array}{ll} k(x_{i}-a{)}^{m}, & x_{i}>a\\ 0, & a\leq x_{i}\leq a\\ k(-x_{i}-a{)}^{m}, & x_{i}<-a \end{array}\right.$	0	[−100, 100]
F21	Multimodal	Penalized 2	$f_{21}(x)=\sum_{i=1}^{dim-1}\;(x_{i}-1{)}^{2}\left[1+\sin^{2}\left(3\pi x_{i+1}\right)\right]+0.1\left\{{sin}^{2}(3\pi x_{1})\right.+(x_{dim}-1{)}^{2}[1+{sin}^{2}(2\pi x_{dim})]\}+\sum_{i=1}^{dim}\;u(x_{i},5,100,4)$	0	[−100, 100]
F22	Fixed	Schwefel	$f_{22}(x)=\sum_{i=1}^{dim}\;x_{i}sin(\sqrt{\left|x_{i}\right|})$	0	[−100, 100]
F23	Multimodal	Lévy	$f_{23}(x)={\sum_{i=1}^{dim}\;(x_{i}-1{)}^{2}[1+{sin}^{2}(3\pi x_{i+1})]+sin}^{2}(3\pi x_{1})+(x_{dim}-1{)}^{2}[1+{sin}^{2}(2\pi x_{dim})]$	0	[−10, 10]
F24	Multimodal	Weierstrass	$f_{24}(x)=\sum_{i=1}^{dim}\;\left(\sum_{k=0}^{k_{max}}\;a^{k}cos(2\pi b^{k}({0.5+x}_{i}))\right)-dim\left(\sum_{k=0}^{k_{max}}\;a^{k}cos(\pi b^{k})\right),a=0.5,b=3,k_{max}=20$	0	[−0.5, 0.5]
F25	Fixed	Solomon	$f_{25}(x)=1+0.1\sqrt{\sum_{i=1}^{dim}\;x_{i}^{2}}-cos\left(2\pi \sqrt{\sum_{i=1}^{dim}\;x_{i}^{2}}\right)$	0	[−100,100]
F26	Fixed	Bohachevsky	$f_{26}(x)=\sum_{i=1}^{dim}\;\left(3x_{i}^{2}-0.3cos(3\pi x_{i})\right)$	0	[−10,10]

**Table 3:** The best fitness, average fitness, standard deviation, average computational time, and average ranking based on average fitness for the 14 algorithms across the 26 test functions.

Function /Metric	AGG-GCRA	GCRA	FDB-AGSK	PSOBOA	WOA	AOA	BOA	NSM-BO	PSO	Pattern Search	GOOSE	LHS-PLS	DE	ACO
F1 Best	0	0	0	0	0	0	0	1.9399 × 10^−6^	1.0903	14229.3021	5.4495 × 10^−3^	20,167.2263	23,191.3365	36,233.1474
F1 Mean	0	0	0	0	0	0	0	1.1685	2.2113	19416.8255	20.3028	26,421.7687	35,676.6206	42,226.9578
F1 Std	0	0	0	0	0	0	0	4.0127	1.0358	2973.2959	84.1605	4064.5578	5066.4294	2908.8408
F1 Time(s)	0.0247	0.0174	5.3131 × 10^−3^	1.6310 × 10^−2^	0.0096	1.9274 × 10^−2^	1.4206 × 10^−2^	0.2248	0.0147	0.2968	1.9789 × 10^−2^	4.2188	0.0018	0.5168
F1 Rank	1	1	1	1	1	1	1	8	9	11	10	12	13	14
F2 Best	0	0	0	0	0	0	0	1.5375 × 10^−4^	2.4146	38.3829	0.533	1637.2499	78.1018	90.4445
F2 Mean	0	0	0	0	0	0	2.1331 × 10^−8^	3.2326 × 10^−3^	4.4176	42.7817	2,788,028.52	285,787,973	1,302,772.22	1,468,718.161
F2 Std	0	0	0	0	0	0	0	3.7095 × 10^−3^	1.2688	4.7118	15,269,285.3	603,192,774	6,926,041.50	2,437,197.031
F2 Time(s)	2.6266 × 10^−2^	1.8048 × 10^−2^	5.9280 × 10^−3^	1.7815 × 10^−2^	0.0109	2.0043 × 10^−2^	1.4920 × 10^−2^	0.2256	0.0151	0.1487	2.0100 × 10^−2^	0.0441	1.2786 × 10^−3^	0.5427
F2 Rank	1	1	1	1	1	1	7	8	9	10	13	14	11	12
F3 Best	0	0	141.6436	0	69.6426	0	0	0.3834	48.6593	386.6074	0.7112	349.8473	436.6124	349.3299
F3 Mean	0	0	426.2177	0	445.8555	0	0	4.1764	83.3512	525.225	2.3813	416.7211	802.3345	491.1673
F3 Std	0	0	123.8602	0	151.7218	0	0	3.3173	21.6652	150.5365	0.9656	58.5605	158.7745	64.7969
F3 Time(s)	0.3612	0.3123	5.3404 × 10^−2^	0.1123	5.7816 × 10^−2^	6.6523 × 10^−2^	0.1103	0.2804	6.1761 × 10^−2^	0.2264	6.8623 × 10^−2^	0.0377	2.9051 × 10^−3^	0.6103
F3 Rank	1	1	10	1	11	1	1	7	8	13	6	9	14	12
F4 Best	0	0	0	0	0.2523	0	2.0495 × 10^−8^	1.4193	1.5195	2.2582 × 10^−4^	0.1236	6.0889	7.8501	6.181
F4 Mean	0	0	3.9441	0	5.053	0	2.6646 × 10^−8^	2.5733	1.9559	5.3745 × 10^−4^	0.2763	6.2894	8.5353	7.095
F4 Std	0	0	3.8346	0	3.1573	0	0	0.5715	0.2275	2.5594 × 10^−4^	0.205	0.2028	0.3507	0.3177
F4 Time(s)	2.66 × 10^−2^	0.0183	5.3560 × 10^−3^	1.6477 × 10^−2^	1.0274 × 10^−2^	1.9173 × 10^−2^	1.4509 × 10^−2^	0.2212	1.4931 × 10^−2^	0.1415	1.9799 × 10^−2^	0.034	1.1742 × 10^−3^	0.5586
F4 Rank	1	1	10	1	11	1	5	9	8	6	7	12	14	13
F5 Best	0	0	5.3031 × 10^−2^	4.7444	2.0897 × 10^−2^	3.9269	4.5202	6.5048 × 10^−8^	0.8884	3.1374 × 10^−7^	3.7242 × 10^−3^	280.2907	248.8288	294.9828
F5 Mean	0	0	0.4956	6.2109	0.1035	5.3097	5.3559	1.1256 × 10^−2^	2.7373	4.7814 × 10^−6^	0.0101	305.505	343.4264	409.294
F5 Std	0	0	0.3105	0.4049	7.3053 × 10^−2^	0.5538	0.4732	3.7013 × 10^−2^	1.6766	6.5110 × 10^−6^	0.0039	25.8801	51.6753	41.9994
F5 Time(s)	2.3279 × 10^−2^	0.0182	0.0052	1.5982 × 10^−2^	1.0183 × 10^−2^	1.8761 × 10^−2^	1.3310 × 10^−2^	0.2223	1.4530 × 10^−2^	0.14	0.0196	0.0243	1.1875 × 10^−3^	0.5453
F5 Rank	1	1	7	11	6	9	10	5	8	3	4	12	13	14
F6 Best	0	4.3524 × 10^−7^	3.9866 × 10^−6^	1.1820 × 10^−5^	4.0721 × 10^−5^	2.6791 × 10^−5^	6.1079 × 10^−4^	4.4489 × 10^−2^	4.0649	0.1879	0.0521	15.8893	20.5087	26.1925
F6 Mean	0	4.1875 × 10^−6^	8.2589 × 10^−4^	2.2505 × 10^−4^	2.1290 × 10^−3^	6.8253 × 10^−4^	2.0296 × 10^−3^	0.1149	15.8092	0.3004	0.1278	22.0865	40.4245	47.8157
F6 Std	0	4.8947 × 10^−6^	2.7612 × 10^−3^	1.5498 × 10^−4^	2.4085 × 10^−3^	5.8479 × 10^−4^	1.0059 × 10^−3^	3.8165 × 10^−2^	10.4808	0.1341	0.0412	4.1589	12.7544	8.1893
F6 Time(s)	0.2542	0.1774	4.1547 × 10^−2^	0.0882	0.0455	5.4340 × 10^−2^	8.6195 × 10^−2^	0.2548	0.0498	0.1332	5.6226 × 10^−2^	0.0282	2.4504 × 10^−3^	0.594
F6 Rank	1	2	5	3	7	4	6	8	11	10	9	12	13	14
F7 Best	0	0	0	0	0	0	0	0	0	0	0	0	0	0
F7 Mean	0	2.0136 × 10^−7^	0	0	0	0	0	0	0	0	0	0	0	0
F7 Std	0	1.0679 × 10^−6^	0	0	0	0	0	0	0	0	0	0	0	0
F7 Time(s)	3.6708 × 10^−2^	0.0267	5.1364 × 10^−3^	1.5876 × 10^−2^	9.7843 × 10^−3^	0.0185	0.0132	0.2171	1.4304 × 10^−2^	0.0134	1.9878 × 10^−2^	0.0338	0.0012	0.5516
F7 Rank	1	14	1	1	1	1	1	1	1	1	1	1	1	1
F8 Best	0	0	0	0	0	0	0	0	1.5873 × 10^−2^	0	2.8639 × 10^−6^	8.1776 × 10^−3^	8.5020 × 10^−2^	4.2330 × 10^−2^
F8 Mean	0	0	0	0	0	0	0	0	0.2286	0	1.2779 × 10^−5^	1.5291 × 10^−2^	0.2975	0.1015
F8 Std	0	0	0	0	0	0	0	0	0.2233	0	7.5423 × 10^−6^	5.2097 × 10^−3^	0.1365	3.3335 × 10^−2^
F8 Time(s)	0.033	0.0212	3.1039 × 10^−2^	0.0764	3.8073 × 10^−2^	4.4416 × 10^−2^	7.6715 × 10^−2^	0.2564	0.046	0.1167	0.0494	0.0333	2.2327 × 10^−3^	0.5852
F8 Rank	1	1	1	1	1	1	1	1	13	1	10	11	14	12
F9 Best	0	0	0	0	0	0	0	2.9706 × 10^−6^	8.9297	1538.8794	0.2115	3276.281	3128.3395	3870.0176
F9 Mean	0	0	0	0	0	0	0	0.1068	27.353	3650.7722	0.9561	3507.6465	4541.3618	5647.2092
F9 Std	0	0	0	0	0	0	0	0.4034	11.5362	2527.0202	0.934	186.6183	648.8771	509.1589
F9 Time(s)	2.7421 × 10^−2^	1.8986 × 10^−2^	4.9784 × 10^−3^	1.5623 × 10^−2^	9.6762 × 10^−3^	1.8831 × 10^−2^	0.0131	0.2201	0.0143	0.1396	1.9342 × 10^−2^	0.0339	1.2213 × 10^−3^	0.5506
F9 Rank	1	1	1	1	1	1	1	8	10	12	9	11	13	14
F10 Best	0	0	28.4817	28.9152	27.1509	28.6782	28.8321	17.4889	312.084	27.8329	26.2799	443,156.311	366,499.537	776,195.6054
F10 Mean	0	0	28.712	28.9617	27.7387	28.8545	28.9063	125.1043	824.2823	2922.7375	84.9944	564,518.114	1,112,877.96	1,294,452.428
F10 Std	0	0	6.8177 × 10^−2^	2.1486 × 10^−2^	0.4435	8.5553 × 10^−2^	3.4005 × 10^−2^	88.9557	268.2789	5332.3357	88.684	148,774.305	385,312.034	186,334.3659
F10 Time(s)	1.8567	1.7819	1.0809 × 10^−2^	2.7269 × 10^−2^	1.5192 × 10^−2^	2.3847 × 10^−2^	2.4665 × 10^−2^	0.2337	0.0197	0.1495	2.5653 × 10^−2^	0.0263	1.4139 × 10^−3^	0.5583
F10 Rank	1	1	4	7	3	5	6	9	10	11	8	12	13	14
F11 Best	0	0	0	0	0	0	0	4.1308 × 10^−6^	39.5478	1894.5796	7.0951 × 10^−2^	5708.8348	6581.6436	8724.161
F11 Mean	0	0	0	0	0	0	0	3.1196 × 10^−2^	120.5195	4949.618	0.1676	7220.6332	13817.1965	15909.7282
F11 Std	0	0	0	0	0	0	0	7.3861 × 10^−2^	63.209	2266.4649	4.9575 × 10^−2^	1337.4239	4371.8978	2421.5444
F11 Time(s)	0.0982	8.3352 × 10^−2^	0.0399	0.0859	0.044	0.0536	0.0841	0.261	4.8942 × 10^−2^	0.1893	5.4641 × 10^−2^	0.0316	2.4947 × 10^−3^	0.5855
F11 Rank	1	1	1	1	1	1	1	8	10	11	9	12	13	14
F12 Best	0	0	0.6695	0.9898	0.6667	0.6756	0.9426	0.6744	69.4898	0.3427	0.8492	12,142.0846	11,518.7959	34,151.5626
F12 Mean	0	1.9982 × 10^−4^	0.767	0.9959	0.6669	0.8505	0.9725	3.1722	231.0978	2.483	1.7969	22,449.9028	43,256.9561	44,770.0338
F12 Std	0	2.6125 × 10^−4^	0.1199	3.1627 × 10^−3^	1.3003 × 10^−4^	0.1075	9.3781 × 10^−3^	1.8195	139.3265	2.5733	1.1575	7977.6302	13097.0083	5430.0965
F12 Time(s)	0.0317	0.0207	0.0052	1.5725 × 10^−2^	9.9140 × 10^−3^	1.8636 × 10^−2^	1.3556 × 10^−2^	0.2236	0.0146	0.1378	0.0197	0.0292	1.1668 × 10^−3^	0.5524
F12 Rank	1	2	4	7	3	5	6	10	11	9	8	12	13	14
F13 Best	25.2111	1078.4966	0	0	0	0	0	0	0	0	8.3514 × 10^−7^	6.9049 × 10^−4^	0.0061	4.0105 × 10^−2^
F13 Mean	137.7624	14,589,152.5	0	0	0	0	0	0	0	0	1.2301 × 10^−3^	0.1082	519.2595	93.7392
F13 Std	109.3553	32,824,190.3	0	0	0	0	0	0	0	0	2.9873 × 10^−3^	0.1624	745.2069	141.3684
F13 Time(s)	3.8810 × 10^−2^	2.2246 × 10^−2^	3.5922 × 10^−2^	7.7428 × 10^−2^	3.9963 × 10^−2^	0.0496	0.0756	0.2682	4.5103 × 10^−2^	0.0319	5.0539 × 10^−2^	0.0359	2.6594 × 10^−3^	0.5848
F13 Rank	12	14	1	1	1	1	1	1	1	1	9	10	13	11
F14 Best	0	0	0	0	0	0	0	0	0	0	0.3861	5.2395	5.7217	0.783
F14 Mean	0	0	0	0	0	0	0	0	0	0	1947.8304	27.0739	1077.7608	705.1322
F14 Std	0	0	0	0	0	0	0	0	0	0	2008.2646	14.897	1412.7496	708.58
F14 Time(s)	0.0261	0.0198	5.0980 × 10^−3^	1.5748 × 10^−2^	0.0097	0.0187	1.3206 × 10^−2^	0.2302	0.0147	0.0357	1.9516 × 10^−2^	0.0293	0.0012	0.5495
F14 Rank	1	1	1	1	1	1	1	1	1	1	14	11	13	12
F15 Best	0	0	0	0	0	0	0	0	0	4.5099 × 10^−7^	3.2750 × 10^−7^	1.8192 × 10^−2^	4.7326 × 10^−3^	0.6389
F15 Mean	0	0	0	0	0	0	0	0	0	8.5054 × 10^−6^	6.3075 × 10^−3^	0.1467	14.6566	29.034
F15 Std	0	0	0	0	0	0	0	0	0	7.6659 × 10^−6^	1.0058 × 10^−2^	0.1302	28.8666	43.1852
F15 Time(s)	2.9519 × 10^−2^	0.02	3.6948 × 10^−2^	0.0797	4.1501 × 10^−2^	0.0507	7.7772 × 10^−2^	0.2596	4.5894 × 10^−2^	0.0358	5.1558 × 10^−2^	0.0297	2.3669 × 10^−3^	0.6228
F15 Rank	1	1	1	1	1	1	1	1	1	10	11	12	13	14
F16 Best	0	0	0	0	0	0	0	3.9833	91.1099	70.3276	109.4377	251.0689	297.7145	297.6154
F16 Mean	0	0	0	0	0	21.4477	44.8648	9.0016	169.7599	122.5299	161.6984	259.8313	337.4773	344.6979
F16 Std	0	0	0	0	0	54.8148	82.7902	3.2511	32.5259	32.9449	41.9444	6.5579	17.8172	17.3044
F16 Time(s)	3.6488 × 10^−2^	2.1836 × 10^−2^	6.5039 × 10^−3^	2.4299 × 10^−2^	0.0118	2.1001 × 10^−2^	0.0256	0.2331	2.1004 × 10^−2^	0.1421	2.5895 × 10^−2^	0.0245	1.4741 × 10^−3^	0.5664
F16 Rank	1	1	1	1	1	7	8	6	11	9	10	12	13	14
F17 Best	0	0	0	0	0	0	0	4.0287	70.4744	21	155.0034	258.3868	257.7023	248.8714
F17 Mean	0	0	0	1.0507 × 10^−8^	11.1028	49.2463	115.955	9.4965	176.7935	42.1495	206.8601	281.0974	311.2447	305.4808
F17 Std	0	0	0	3.6072 × 10^−8^	43.3103	60.8974	77.7255	2.8768	38.4849	18.079	30.806	15.6325	26.6802	18.2432
F17 Time(s)	0.0301	0.0221	6.8767 × 10^−3^	3.2395 × 10^−2^	1.3646 × 10^−2^	2.3441 × 10^−2^	3.1329 × 10^−2^	0.2307	2.3152 × 10^−2^	0.1318	0.0276	0.0352	0.0016	0.5648
F17 Rank	1	1	1	4	6	8	9	5	10	7	11	12	14	13
F18 Best	0	0	0	0	0	0	1.9024 × 10^−8^	1.9620 × 10^−3^	1.211	7.7482	5.2772 × 10^−2^	17.3383	15.0377	16.5686
F18 Mean	0	0	0	0	0	0	2.7516 × 10^−8^	0.756	2.4328	12.5622	6.3955	17.592	16.2281	17.2635
F18 Std	0	0	0	0	0	0	0	0.7139	0.4835	2.8488	7.4417	0.2121	0.6602	0.2797
F18 Time(s)	3.9665 × 10^−2^	2.4574 × 10^−2^	6.8717 × 10^−3^	2.0754 × 10^−2^	0.0122	2.0866 × 10^−2^	1.9857 × 10^−2^	0.2399	0.0216	0.1509	0.0262	0.026	1.6078 × 10^−3^	0.5749
F18 Rank	1	1	1	1	1	1	7	8	9	11	10	14	12	13
F19 Best	0	0	0	0	0	0	0	1.7373 × 10^−2^	5.5459 × 10^−2^	44.4994	9.7781 × 10^−4^	251.4396	217.33	276.6861
F19 Mean	0	0	6.1988 × 10^−2^	0	4.6423 × 10^−3^	5.3737 × 10^−3^	0	0.2573	0.1391	163.32	255.7776	277.7318	316.6874	371.6827
F19 Std	0	0	0.236	0	2.5427 × 10^−2^	2.9433 × 10^−2^	0	0.2165	5.3178 × 10^−2^	73.5493	216.7762	22.1084	41.3534	34.9386
F19 Time(s)	0.1623	0.115	0.0116	2.7403 × 10^−2^	1.6550 × 10^−2^	0.027	2.7819 × 10^−2^	0.2399	2.6147 × 10^−2^	0.1536	2.9002 × 10^−2^	0.0267	0.0017	0.5777
F19 Rank	1	1	7	1	5	6	1	9	8	10	11	12	13	14
F20 Best	0	0	0	0	0	0	0	4.4821 × 10^−5^	2.0752	1.2147	4.7314	16.8585	35.6176	35.7455
F20 Mean	0	4.1074 × 10^−7^	0	0	0.6393	0	0	3.2476 × 10^−3^	5.6527	6.3539	7.2314	21.2742	46.2738	44.1548
F20 Std	0	2.1893 × 10^−6^	0	0	3.5015	0	1.2303 × 10^−8^	6.5164 × 10^−3^	2.2916	5.2892	2.0369	3.3503	4.0218	2.973
F20 Time(s)	0.0231	0.0198	6.4092 × 10^−3^	1.8716 × 10^−2^	1.1262 × 10^−2^	2.0097 × 10^−2^	2.3484 × 10^−2^	0.2325	1.9482 × 10^−2^	0.1495	2.4172 × 10^−2^	0.0255	1.4180 × 10^−3^	0.5768
F20 Rank	1	6	1	1	8	1	5	7	9	10	11	12	14	13
F21 Best	0	0	2.1148 × 10^−3^	0.5079	3.0826 × 10^−3^	0.3587	0.2319	0	8.6050 × 10^−3^	0	1.7544	6.5647	8.2759	10.3408
F21 Mean	0	2.8567 × 10^−7^	2.9429 × 10^−2^	0.9424	1.1607 × 10^−2^	0.6078	0.4724	1.7299 × 10^−2^	4.2368 × 10^−2^	1.3318 × 10^−6^	3.9486	8.8072	13.4582	12.6119
F21 Std	0	4.3539 × 10^−7^	3.0725 × 10^−2^	0.1709	8.9169 × 10^−3^	0.1962	0.1309	6.1369 × 10^−2^	3.1413 × 10^−2^	2.1931 × 10^−6^	1.1765	1.4282	2.1139	1.1492
F21 Time(s)	1.9423	1.7516	0.0965	0.1995	0.1	0.1084	0.1971	0.3318	0.1064	0.2921	0.111	0.0479	0.0045	0.6564
F21 Rank	1	2	6	10	4	9	8	5	7	3	11	12	14	13
F22 Best	0	0	3.5834 × 10^−3^	2.1566	0.048	1.6618	2.005	1.3087 × 10^−8^	0.2014	0.011	2.6443 × 10^−3^	14.3253	9.0591	10.2405
F22 Mean	0	6.6016 × 10^−7^	0.1033	2.8489	0.1379	2.6876	2.7511	6.2585 × 10^−3^	0.5728	1.0989 × 10^−2^	1.2149 × 10^−2^	17.6382	13.679	15.704
F22 Std	0	1.2625 × 10^−6^	0.1066	0.1957	7.5121 × 10^−2^	0.3906	0.3166	1.2020 × 10^−2^	0.2049	2.0861 × 10^−6^	1.4515 × 10^−2^	2.4804	1.8625	1.5966
F22 Time(s)	1.7935	1.5898	0.0989	0.1989	0.1018	0.1109	0.2019	0.3374	0.1086	0.294	0.1133	0.0371	0.0046	0.6562
F22 Rank	1	2	6	11	7	9	10	3	8	4	5	14	12	13
F23 Best	0	0	6.2351 × 10^−4^	6.1177	1.2115 × 10^−2^	6.0541	6.4779	0.0124	0.6915	1.2395 × 10^−2^	0.158	2.847	8.1607	12.1094
F23 Mean	0	1.9215 × 10^−4^	0.3656	15.9904	0.3676	10.2128	12.6304	0.0544	5.6636	2.3468 × 10^−2^	0.6944	4.1375	14.715	14.6409
F23 Std	0	3.3954 × 10^−4^	0.4608	4.0057	0.4447	1.8923	2.2726	7.6478 × 10^−2^	4.1001	1.7110 × 10^−2^	0.5712	1.2498	2.599	1.6099
F23 Time(s)	1.3545	1.2704	1.2930 × 10^−2^	0.0312	0.0182	2.6490 × 10^−2^	0.0336	0.2377	0.0243	0.1543	0.0295	0.0271	1.6134 × 10^−3^	0.5618
F23 Rank	1	2	5	14	6	10	11	4	9	3	7	8	13	12
F24 Best	0	0	0	0	0	0	0	0	2.3174 × 10^−4^	1.2069	0	34.7618	14.3067	39.3755
F24 Mean	0	0	0	7.8268 × 10^−5^	0	5.7624	1.6588	4.9605 × 10^−4^	4.8583	1.779	7.8172	40.0183	18.408	42.6382
F24 Std	0	0	0	1.7532 × 10^−4^	0	8.3353	3.0844	2.6863 × 10^−3^	3.8653	0.6616	6.4364	3.3385	2.2842	1.0708
F24 Time(s)	1.5564	1.256	1.073	2.0872	1.0772	1.0734	2.1138	1.3639	1.106	1.7033	1.0881	0.1989	3.8840 × 10^−2^	1.5972
F24 Rank	1	1	1	5	1	10	7	6	9	8	11	13	12	14
F25 Best	0	0	0	9.9496 × 10^−2^	0	9.9496 × 10^−2^	0.3981	2.4874	0.8955	36.4958	0.8955	97.7456	108.255	135.0328
F25 Mean	0	0	0.1526	9.9497 × 10^−2^	0.1658	9.9496 × 10^−2^	0.8459	5.4163	1.716	57.7653	1.5654	129.9475	145.4765	170.0775
F25 Std	0	0	0.1425	2.3512 × 10^−6^	0.1835	7.3556 × 10^−7^	0.1513	1.752	0.3619	29.1443	0.4072	22.35	22.63	12.2997
F25 Time(s)	3.5189 × 10^−2^	2.0889 × 10^−2^	6.0106 × 10^−3^	1.8021 × 10^−2^	1.0965 × 10^−2^	1.9399 × 10^−2^	1.5407 × 10^−2^	0.2293	1.5514 × 10^−2^	0.1423	2.0651 × 10^−2^	0.0271	1.2769 × 10^−3^	0.5573
F25 Rank	1	1	5	4	6	3	7	10	9	11	8	12	13	14
F26 Best	0	0	0	0	0	0	0	1.5132 × 10^−6^	16.4149	63.8909	1.4907	135.6216	186.4904	253.5763
F26 Mean	0	0	0	0	0	0	0	0.5355	24.0663	97.2697	4.6319	234.5143	276.4403	323.3349
F26 Std	0	0	0	0	0	0	0	1.2113	5.0202	34.1846	2.0569	56.6051	38.8565	25.8319
F26 Time(s)	2.4409 × 10^−2^	1.9670 × 10^−2^	7.4357 × 10^−3^	2.0204 × 10^−2^	1.3020 × 10^−2^	0.0214	2.1382 × 10^−2^	0.2312	2.6075 × 10^−2^	0.1467	2.9143 × 10^−2^	0.033	1.7073 × 10^−3^	0.5669
F26 Rank	1	1	1	1	1	1	1	8	10	11	9	12	13	14
Paired rank +/=/−		8/18/0	15/10/1	11/14/1	10/15/1	12/13/1	13/12/1	21/4/1	22/3/1	24/1/1	24/1/1	24/1/1	23/2/1	25/1/0
Avg. rank	1.42	2.38	3.23	3.54	3.69	3.81	4.73	6.00	8.08	8.38	8.92	11.38	12.58	12.77
Overall rank	1	2	3	4	5	6	7	8	9	10	11	12	13	14

**Table 4:** Friedman ranking scores of 14 algorithms.

Algorithms	Friedman Scores	Rank
**AGG-GCRA**	1.7308	**1**
GCRA	3.3077	2
BOA	7.7692	8
WOA	5.1154	4
GOOSE	8.8846	11
AOA	5.2308	5
PSO	8.8462	10
DE	12.8462	13
ACO	13.0769	14
NSM-BO	6.6538	7
PSOBOA	6.5385	6
FDB-AGSK	4.9231	3
LHS-PLS	11.6154	12
Patternsearch	8.4615	9

**Table 5:** Wilcoxon signed-rank test results for the AGG-GCRA compared to the other 13 algorithms.

Algorithms	Wilcoxon Test *p*-Value	Significant
AGG-GCRA-GCRA	1.3183 × 10^−4^	Yes
AGG-GCRA-BOA	9.6755 × 10^−5^	Yes
AGG-GCRA-WOA	1.5487 × 10^−3^	Yes
AGG-GCRA-GOOSE	7.0443 × 10^−5^	Yes
AGG-GCRA-AOA	2.9531 × 10^−4^	Yes
AGG-GCRA-PSO	7.0443 × 10^−5^	Yes
AGG-GCRA-DE	9.3386 × 10^−6^	Yes
AGG-GCRA-ACO	9.3386 × 10^−6^	Yes
AGG-GCRA-NSM-BO	4.6925 × 10^−4^	Yes
AGG-GCRA-PSOBOA	3.7291 × 10^−4^	Yes
AGG-GCRA-FDB-AGSK	2.3556 × 10^−3^	Yes
AGG-GCRA-LHS-PLS	1.8726 × 10^−5^	Yes
AGG-GCRA-Patternsearch	4.1000 × 10^−5^	Yes

**Table 6:** Optimal values, corresponding design variables, and ACTs of 12 algorithms on the weight minimization of a speed reducer problem.

Algorithm	Optimal Value	X1	X2	X3	X4	X5	X6	X7	ACTs
AGG-GCRA	2994.2343	3.499	0.7	17	7.3	7.7152	3.3505	5.2867	0.2735
GCRA	2994.2343	3.499	0.7	17	7.3	7.7152	3.3505	5.2867	0.2276
BOA	3129.3275	3.6	0.7	17	8.0137	8.056	3.3529	5.4114	0.3277
WOA	3008.3606	3.4992	0.7	17	7.3	8.067	3.3752	5.2868	0.166
GOOSE	2998.1153	3.5007	0.7	17	7.4365	7.8026	3.3516	5.2867	0.1863
AOA	3007.0453	3.4921	0.7	17	7.3	7.7122	3.3583	5.2867	0.1636
PSO	2994.2343	3.499	0.7	17	7.3	7.7152	3.3505	5.2867	0.1949
DE	3028.0132	3.506	0.7	17	8.3	8.2652	3.3825	5.2897	0.0066
ACO	3148.4498	3.56	0.7119	17.0492	8.1819	7.9502	3.5407	5.2887	0.2844
NSM-BO	2994.2343	3.499	0.7	17	7.3	7.7152	3.3505	5.2867	0.4035
PSOBOA	3067.0392	4.5055	1.7	18	8.9677	9.0821	4.3938	6.3556	0.3273
FDB-AGSK	2994.2364	3.499	0.7	17	7.3	7.7152	3.3505	5.2867	0.1024

**Table 7:** Optimal values, mean, median, standard deviation, and ranking of 12 algorithms on the weight minimization of a speed reducer problem.

Algorithm	Best	Mean	Worst	Median	Std	Rank
AGG-GCRA	2994.2343	2994.2343	2994.2343	2994.2343	0	**1**
GCRA	2994.2343	3081.6584	3187.6037	3068.9192	47.2691	6
BOA	3129.3275	5144.0961	24,197.8576	3796.02	4608.6811	11
WOA	3008.3606	3330.8251	5331.2281	3128.3262	527.3161	9
GOOSE	2998.1153	3033.1401	3500.4813	3007.9772	110.0764	5
AOA	3007.0453	5720.7911	13,313.9628	4494.0398	2918.6664	12
PSO	2994.2343	3001.0945	3033.7004	2994.2343	14.3376	4
DE	3028.0132	3112.3207	3399.8135	3085.2303	87.0902	7
ACO	3148.4498	3281.7124	3493.0759	3270.6093	92.2613	8
NSM-BO	2994.2343	2994.2343	2994.2343	2994.2343	0	1
PSOBOA	3067.0392	5036.9274	18,460.9648	3296.2795	4299.0119	10
FDB-AGSK	2994.2364	2994.5136	2996.9713	2994.3043	0.6662	3

**Table 8:** Optimal values, corresponding design variables, and ACTs of 12 algorithms on the spring design problem.

Algorithm	Optimal Value	d	D	N	ACTs
AGG-GCRA	0.0127	0.0521	0.3671	10.7045	0.239
GCRA	0.0128	0.05	0.3172	14.1083	0.2164
BOA	0.0134	0.0521	0.3671	10.7045	0.2876
WOA	0.0127	0.0521	0.3671	10.7045	0.1451
GOOSE	0.0127	0.0521	0.3671	10.7045	0.152
AOA	0.0127	0.0521	0.3671	10.7045	0.141
PSO	0.0127	0.0521	0.3671	10.7045	0.1734
DE	0.0132	0.05	0.3105	15	0.0058
ACO	0.0138	0.0543	0.4041	9.528	0.1868
NSM-BO	0.0127	0.0521	0.3671	10.7045	0.3903
PSOBOA	0.0168	0.1259	1.3967	10.7521	0.2882
FDB-AGSK	0.0127	0.0521	0.3671	10.7045	0.0886

**Table 9:** Optimal values, mean, median, standard deviation, and ranking of 12 algorithms on the spring design problem.

Algorithm	Best	Mean	Worst	Median	Std	Rank
AGG-GCRA	0.0127	0.0127	0.0127	0.0127	0	**1**
GCRA	0.0128	0.0133	0.0156	0.0132	0.0006	6
BOA	0.0134	212.8719	2251.7441	0.0225	573.477	10
WOA	0.0127	0.0132	0.0145	0.0132	0.0005	5
GOOSE	0.0127	0.013	0.0141	0.0128	0.0004	4
AOA	0.0127	1387.6809	27,743.633	0.0135	6203.5478	11
PSO	0.0127	0.0134	0.0156	0.0131	0.0009	7
DE	0.0132	0.0185	0.0344	0.0174	0.005	9
ACO	0.0138	0.018	0.0237	0.0175	0.0029	8
NSM-BO	0.0127	0.0127	0.0127	0.0127	0	**1**
PSOBOA	0.0168	26,132.2492	82,511.1867	17,171.2997	30,481.7	12
FDB-AGSK	0.0127	0.0127	0.0128	0.0127	0	**1**

**Table 10:** Optimal values, corresponding design variables, and ACTs of 12 algorithms on the welded beam design problem.

Algorithm	Optimal Value	h	l	t	b	ACTs
AGG-GCRA	1.6702	0.1988	3.3373	9.192	0.1988	0.3286
GCRA	1.6702	0.1988	3.3373	9.192	0.1988	0.2971
BOA	2.2363	0.125	6.0015	8.3417	0.2657	0.3357
WOA	1.788	0.2012	3.4429	8.6622	0.2248	0.1704
GOOSE	1.7436	0.2079	3.2831	8.8037	0.2168	0.1784
AOA	1.7853	0.1356	5.1079	9.184	0.1992	0.1663
PSO	1.6702	0.1988	3.3373	9.192	0.1988	0.2009
DE	2.0497	0.2083	4.836	8.6484	0.2319	0.0067
ACO	1.8356	0.1757	3.768	9.8449	0.2028	0.2329
NSM-BO	1.6702	0.1988	3.3373	9.192	0.1988	0.4244
PSOBOA	2.2385	0.875	5.7481	7.3978	1.1241	0.3362
FDB-AGSK	1.6703	0.1989	3.3368	9.192	0.1988	0.0987

**Table 11:** Optimal values, mean, median, standard deviation, and ranking of 12 algorithms on the welded beam design problem.

Algorithm	Best	Mean	Worst	Median	Std	Rank
AGG-GCRA	1.6702	1.6718	1.7012	1.6702	0.0511	1
GCRA	1.6702	1.7347	1.782	1.7345	0.0216	5
BOA	2.2363	2.8197	3.4998	2.7493	0.3204	11
WOA	1.788	2.5983	4.7071	2.3527	0.7257	8
GOOSE	1.7436	2.028	2.6252	2.0319	0.2338	6
AOA	1.7853	3.0729	3.6827	3.1841	0.5627	12
PSO	1.6702	1.7068	1.9185	1.6723	0.0681	4
DE	2.0497	2.6444	3.9293	2.5199	0.4794	10
ACO	1.8356	2.3162	2.8637	2.311	0.256	7
NSM-BO	1.6702	1.6849	1.8167	1.6702	0.0451	3
PSOBOA	2.2385	2.6202	3.2595	2.6091	0.2433	9
FDB-AGSK	1.6703	1.6723	1.6802	1.6714	0.0025	2

**Table 12:** Optimal values, corresponding design variables, and ACTs of 12 algorithms on the GTCD problem.

Algorithm	Optimal Value	L	r	D	ACTs
AGG-GCRA	1,677,759.276	24.469	1.1587	20	0.1985
GCRA	1,677,759.276	24.469	1.1587	20	0.1644
BOA	1,677,905.674	23.9545	1.1382	20	0.169
WOA	1,677,759.276	24.4691	1.1587	20	0.085
GOOSE	1,677,759.276	24.469	1.1587	20	0.0939
AOA	1,677,762.951	24.3611	1.1576	20	0.0828
PSO	1,677,759.276	24.469	1.1587	20	0.115
DE	1,678,411.336	23.5748	1.1109	20	0.0038
ACO	1,911,487.417	26.3278	1.2053	22.5931	0.1507
NSM-BO	1,677,759.276	24.469	1.1587	20	0.3045
PSOBOA	1,685,732.677	21	1.1129	21	0.1701
FDB-AGSK	1,677,759.276	24.469	1.1587	20	0.0641

**Table 13:** Optimal values, mean, median, standard deviation, and ranking of 12 algorithms on the GTCD problem.

Algorithm	Mean	Best	Worst	Median	Std	Rank
AGG-GCRA	1,677,759.276	1,677,759.276	1,677,759.276	1677,759.276	0	**1**
GCRA	1,677,759.276	1,677,759.276	1,677,759.276	1,677,759.276	0	**1**
BOA	1,677,905.674	1,684,415.954	1,685,732.772	1,685,732.68	2746.038	9
WOA	1,677,759.276	1,677,759.276	1,677,759.28	1,677,759.276	0.001	**1**
GOOSE	1,677,759.276	1,717,117.313	2,342,300.295	1,681,853.006	147,350.6514	**1**
AOA	1,677,762.951	1,705,262.898	1,900,901.635	1,685,744.13	57,901.2131	8
PSO	1,677,759.276	1,728,464.905	2,675,925.065	1,677,759.276	223,022.4058	**1**
DE	1,678,411.336	1,844,361.336	2,771,701.929	1,738,970.151	273,878.2691	10
ACO	1,911,487.417	2,109,740.559	2,392,295.085	2,082,094.482	136,205.472	12
NSM-BO	1,677,759.276	1,677,759.276	1,677,759.276	1,677,759.276	0	**1**
PSOBOA	1,685,732.677	1,685,733.183	1,685,736.165	1,685,732.859	0.8409	11
FDB-AGSK	1,677,759.276	1,677,759.276	1,677,759.276	1,677,759.276	0	**1**

**Table 14:** Optimal values, corresponding design variables, and ACTs of 12 algorithms on the three-bar truss design problem.

Algorithm	Optimal Value	X1	X2	ACTs
AGG-GCRA	263.8523	0.7884	0.4081	0.2554
GCRA	263.8537	0.7884	0.4081	0.218
BOA	265.0689	0.7843	0.4202	0.2835
WOA	265.8775	0.789	0.4064	0.1926
GOOSE	263.8524	0.7884	0.4081	0.4691
AOA	264.4814	0.7882	0.4087	0.1346
PSO	263.8525	0.7884	0.4081	0.1695
DE	264.965	0.788	0.4096	0.0056
ACO	264.2652	0.7858	0.4153	0.1618
NSM-BO	263.8523	0.7884	0.4081	0.3629
PSOBOA	267.1286	1.7813	1.1281	0.282
FDB-AGSK	263.8523	0.7884	0.4081	0.0849

**Table 15:** Optimal values, mean, median, standard deviation, and ranking of 12 algorithms on the three-bar truss design problem.

Algorithm	Best	Mean	Worst	Median	Std	Rank
AGG-GCRA	263.8523	263.8523	263.8523	263.8523	0	**1**
GCRA	263.8523	263.8523	263.8537	263.8523	0	**1**
BOA	263.876	264.258	265.0689	264.1948	0.3369	10
WOA	263.8526	264.422	265.8775	264.0941	0.6677	11
GOOSE	263.8523	263.8524	263.8524	263.8523	0	**1**
AOA	263.8524	263.987	264.4814	263.8798	0.2106	12
PSO	263.8523	263.8524	263.8525	263.8523	0	**1**
DE	263.8605	264.0924	264.965	264.045	0.2505	9
ACO	263.8651	263.9623	264.2652	263.9431	0.0979	8
NSM-BO	263.8523	263.8523	263.8523	263.8523	0	**1**
PSOBOA	263.9719	264.8713	267.1286	264.5127	0.8816	7
FDB-AGSK	263.8523	263.8523	263.8523	263.8523	0	**1**

**Table 16:** Optimal values, corresponding design variables, and ACTs of 12 algorithms on the multiple-disk clutch brake design problem.

Algorithm	Optimal Value	X1	X2	X3	X4	X5	ACTs
AGG-GCRA	**0.2352**	70	90	1	1000	2	0.3157
GCRA	**0.2352**	70	90	1	1000	2	0.2874
BOA	0.2523	68.3183	90	1	247.3377	2	0.7765
WOA	**0.2352**	70	90	1	1000	2	0.1887
GOOSE	**0.2352**	70	90	1	1000	2	0.1963
AOA	0.2356	69.969	90	1	469.3174	2	0.1883
PSO	**0.2352**	70	90	1	1000	2	0.2167
DE	0.2363	69.8969	90	1	748.143	2	0.0074
ACO	0.2444	70.2503	90.3606	1.0113	4.3049	2.053	0.2714
NSM-BO	**0.2352**	70	90	1	1000	2	0.4129
PSOBOA	0.2368	68.8458	91	0	48.7086	1	0.3751
FDB-AGSK	**0.2352**	70	90	1	1000	2	0.1074

**Table 17:** Optimal values, mean, median, standard deviation, and ranking of 12 algorithms on the multiple-disk clutch brake design problem.

Algorithm	Best	Mean	Worst	Median	Std	Rank
AGG-GCRA	0.2352	**0.2352**	0.2352	0.2352	0	**1**
GCRA	0.2352	0.2401	0.2617	0.2352	0.0024	7
BOA	0.2523	0.3163	0.3308	0.3308	0.0231	11
WOA	0.2352	**0.2352**	0.2352	0.2352	0	**1**
GOOSE	0.2352	0.2399	0.2543	0.2352	0.0068	6
AOA	0.2356	0.2562	0.3024	0.2522	0.0209	9
PSO	0.2352	**0.2352**	0.2352	0.2352	0	**1**
DE	0.2363	0.2433	0.2609	0.2413	0.0064	8
ACO	0.2444	0.2772	0.3	0.2772	0.0133	10
NSM-BO	0.2352	**0.2352**	0.2352	0.2352	0	**1**
PSOBOA	0.2368	0.3255	0.3308	0.3308	0.021	12
FDB-AGSK	0.2352	**0.2352**	0.2352	0.2352	0	**1**

## Data Availability

All data used and/or analyzed during this research are openly available and can be accessed freely. If needed, they can be requested from the corresponding author.

## Appendix A

### Source and Implementation of the Compared Algorithms

**Algorithms**	**Source/Implementation**
GCRA	https://www.mathworks.com/matlabcentral/fileexchange/165241-greater-cane-rat-algorithm-gcra (Accessed on 10 July 2025)
BOA	https://ieeexplore.ieee.org/abstract/document/9641410 (Accessed on 10 July 2025)
WOA	https://www.mathworks.com/matlabcentral/fileexchange/55667-the-whale-optimization-algorithm (Accessed on 10 July 2025)
GOOSE	https://www.mathworks.com/matlabcentral/fileexchange/163071-goose-algorithm (Accessed on 10 July 2025)
AOA	https://www.researchgate.net/publication/348653867_The_Arithmetic_Optimization_Algorithm_AOA_-_Matlab_Code (Accessed on 10 July 2025)
PSO	https://www.mathworks.com/matlabcentral/fileexchange/52857-particle-swarm-optimization-pso (Accessed on 10 July 2025)
DE	https://www.mathworks.com/matlabcentral/fileexchange/18593-differential-evolution?s_tid=srchtitle (Accessed on 10 July 2025)
ACO	https://www.mathworks.com/matlabcentral/fileexchange/52859-ant-colony-optimization-aco (Accessed on 10 July 2025)
NSM-BO	https://www.mathworks.com/matlabcentral/fileexchange/180062-nsm-bo-algorithm?s_tid=srchtitle (Accessed on 10 July 2025)
PSOBOA	https://www.mathworks.com/matlabcentral/fileexchange/18593-differential-evolution?s_tid=srchtitle (Accessed on 10 July 2025)
FDB-AGSK	https://www.mathworks.com/matlabcentral/fileexchange/129154-fdb-agsk?s_tid=srchtitle (Accessed on 10 July 2025)
LHS-PLS	lhsdesign is from MATLAB Statistics and Machine Learning Toolbox, used to generate Latin Hypercube sample points. https://www.mathworks.com/help/stats/lhsdesign.html (Accessed on 10 July 2025) fminsearch is MATLAB’s built-in Nelder–Mead simplex method for local search. https://www.mathworks.com/help/matlab/ref/fminsearch.html (Accessed on 10 July 2025)
Patternsearch	patternsearch is from MATLAB Global Optimization Toolbox, implementing the MADS (Mesh Adaptive Direct Search) algorithm. https://www.mathworks.com/help/gads/patternsearch.html (Accessed on 10 July 2025)
